# Low-Load endoluminal navigation with a magnetically actuated medical soft microrobot

**DOI:** 10.3389/fmedt.2026.1717944

**Published:** 2026-01-23

**Authors:** Julio Guerra, Andrei Malchikov, Sergey Jatsun, Petr Ryapolov, Andres Santiago Martinez-Leon

**Affiliations:** 1 Facultad de Ingeniería en Ciencias Aplicadas, Universidad Técnica del Norte, Ibarra, Ecuador; 2 Department of Mechanics, Mechatronics and Robotics, South-West State University, Kursk, Russia; 3 Department of Nanotechnologies, Microelectronis, General and Applied Physics, South-West State University, Kursk, Russia; 4 Department of Earth Sciences, Universidad Estatal Amazónica, Pastaza, Ecuador

**Keywords:** autonomousmotion control system, magnetically actuated microrobots, magneticparticle-embedded polymers, minimally invasive medical devices, nonlinear contact dynamics, soft magnetic microrobots

## Abstract

Minimally invasive endoluminal interventions increasingly rely on magnetic actuation to navigate narrow lumens while limiting wall loads. Here we present a contact-aware control framework for steering a deformable, silicone-based soft microrobot with embedded magnetic particles using an externally positioned permanent magnet. We develop a dynamic model capturing viscous drag, nonlinear frictional loads, and viscoelastic wall contact, and implement a closed-loop architecture that combines vision-based state estimation with model-based force inference while optimizing magnet orientation to regulate the force vector and normal reaction. Performance is evaluated in simulation and on a benchtop testbed across three control modes. In the nominal-case benchmark, force-plus-angle control reduced the root-mean-square tracking error from 4.8 to 2.1 mm (−56%), decreased peak tracking error from 14.6 to 8.0 mm (−45%), lowered the integrated performance index from 4.4 × 10^−^¹⁰ to 1.6 × 10^−^¹⁰ (−64%), and attenuated peak normal reaction force from 2.0 × 10^−^^6^ to 0.8 × 10^−^^6^ *N* (−60%) compared with operation without force regulation. To assess robustness, we further performed a simulation-based Monte Carlo analysis (*n* = 500 trials per mode) under parametric uncertainty and measurement noise, confirming that the contact-aware modes preserve their performance advantage; non-parametric tests indicated statistically significant inter-mode differences with moderate-to-large effect sizes. A trade-off analysis in the {tracking error, peak normal load} plane showed that, in the explored regime, improved tracking does not inherently require higher peak contact forces. Finally, a first-order shear-thinning surrogate suggested low sensitivity of the relative conclusions to moderate non-Newtonian effects. Overall, the results identify force-aware magnet orientation as a safety-relevant control degree of freedom for endoluminal navigation and provide a transferable control methodology for future magnetic microrobotic platforms.

## 1 Introduction

Minimally invasive medicine increasingly leverages miniature robotic devices able to reach remote anatomical targets without open surgery. Microrobots promise reduced collateral damage, improved therapeutic precision, and enhanced patient safety by enabling fine maneuvering in confined, delicate environments (1–3, 34, 35). Among the most mature developments are magnetically steerable catheters and remotely actuated devices driven by external magnetic fields, whose superior steerability and motion accuracy lower the risk of vessel and tissue trauma (4, 5, 34).

Beyond tethered tools, wireless microrobots have progressed toward accessing hard-to-reach regions while potentially lowering infection risks and procedure time (6, 7, 34). These devices can be introduced intravascularly or endoluminally for targeted drug delivery, precision therapy, biopsy, or biosensing (8, 9). Clinically plausible scenarios include: (i) localized pharmacological delivery in sub-millimeter ductal or distal vascular branches where conventional catheters struggle; and (ii) gentle micromanipulation in soft lumens (e.g., biliary or urological ducts) where limiting normal forces is essential to avoid abrasion or perforation (34, 36). Recent demonstrations of magnetic microfiberbots for embolization further indicate a viable translational path for magnetically actuated soft microdevices (36).

At micro- to millimeter scales, motion unfolds in low Reynolds number regimes where viscous forces dominate and reciprocal strokes fail to generate net propulsion, making field torques/gradients and non-reciprocal strategies central to steering and thrust (10–12, 35). Performance is strongly modulated by the fluid's rheology (hematocrit, viscoelasticity) and by contact interactions at the wall–device interface, which influence sticking, stalling, and off-axis drift; recent quantitative analyses in blood surrogates corroborate these constraints on drag and maneuverability (13, 34, 37). Consequently, mathematical modeling and closed-loop control that explicitly incorporate fluidic drag, adhesion/friction, and normal forces are indispensable (14, 15, 38).

Because onboard actuation and power scale poorly at these sizes, external magnetic fields remain the dominant strategy. Foundational work explored electromagnets (16) and superconducting/MRI-grade magnets (17, 18) to sculpt magnetic energy landscapes for pulling/steering (15). Contemporary systems complement these with compact permanent-magnet architectures and hybrid arrays that raise force density and portability, widening the feasible clinical footprint (35, 39). On the control side, methods range from linear optimal control (e.g., LQR) and observer-based feedback to model-predictive and learning-based schemes that address nonlinearity and uncertainty (19–24, 38). Yet, many controllers still simplify wall contact and load transfer, despite their central role in safety (limiting tissue stress) and efficacy (preventing sticking and trajectory deviation) (22, 34, 37).

Despite rapid progress in magnetic navigation platforms, a primary barrier to clinical translation remains safe operation in confined lumens where wall contact is unavoidable. In such settings, tracking accuracy and contact load are tightly coupled: insufficient regulation of the normal reaction can induce sticking, off-axis drift, and abrupt contact transients, whereas overly conservative actuation may compromise targeting precision. Therefore, control methods that explicitly model and regulate contact loads while preserving trajectory tracking address a clinically and engineering-relevant bottleneck for endoluminal microrobotic interventions.

The main objective of this study is to develop and experimentally benchmark a contact-aware control methodology for magnetically actuated, soft deformable microrobots navigating confined lumens, in which wall interaction is unavoidable and safety depends on regulating contact loads in addition to trajectory tracking. To achieve this objective, we (i) formulate a comprehensive dynamic model for a deformable spherical magnetoactive object (MO) that integrates viscous drag, friction regimes (sticking, sliding, rolling), and viscoelastic contact; (ii) design a closed-loop control architecture that combines vision-based state estimation with model-based force inference and magnet-orientation optimization to regulate the magnetic force vector and reduce the normal reaction while tracking a desired trajectory; and (iii) validate the approach using a benchtop testbed together with simulation-based robustness and inference analyses, including Monte Carlo uncertainty propagation, non-parametric statistical testing with effect sizes, a Pareto trade-off characterization between tracking and peak contact load, and a first-order sensitivity study to shear-thinning (surrogate) non-Newtonian effects. These contributions position contact-aware force regulation as a safety-relevant degree of freedom for endoluminal magnetic navigation, while clearly delimiting the present work as a bench-validated proof-of-concept that motivates subsequent evaluation in anatomical phantoms and physiological fluids.

The remainder of the paper is organized as follows: Section 2 presents the dynamic model and control architecture, including the force estimation loop and the simulation/robustness protocol; Section 3 reports simulation and bench results, including statistical significance, robustness distributions, and trade-off analyses; Section 4 discusses translational implications, limitations, and future directions.

## 2 Methodology

### 2.1 Mathematical model of a magnetically active object in a viscous medium

In this study, the term “microrobot” refers to a deformable spherical magnetoactive object (MO) with a diameter of 1–2 mm, whose controlled motion is achieved through the magnetic field generated by a permanent magnet. The permanent magnet is positioned using a motorized two-degree-of-freedom electromechanical system, which creates the required magnetic potential landscape for microrobot propulsion (25).

**Figure 1:**
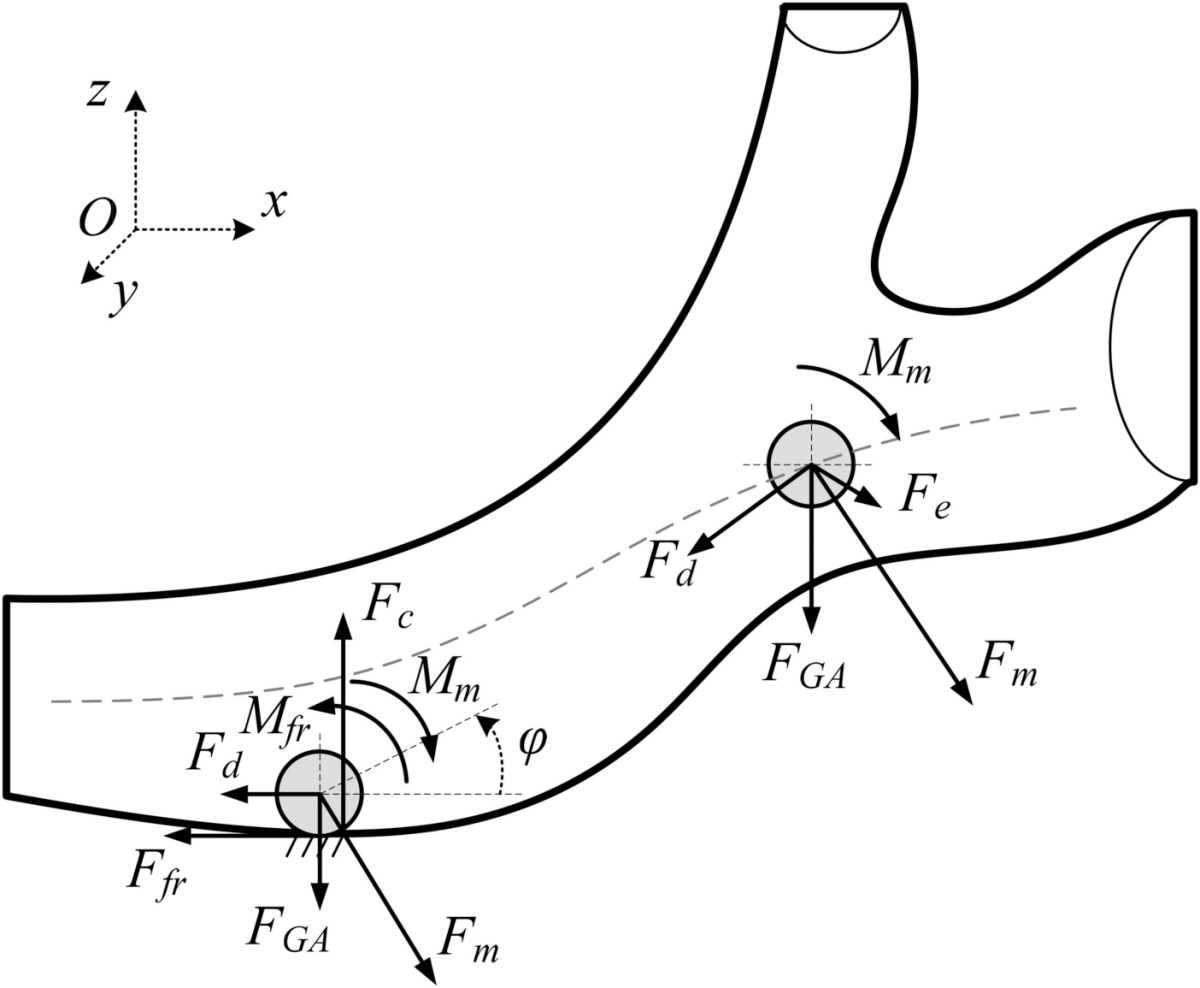
Computational scheme for the magnetoactive object (MO) moving inside a viscous-fluid-filled channel under external permanent-magnet actuation. The model accounts for magnetic forcing, hydrodynamic drag, frictional interaction with the wall, and viscoelastic contact effects.

For mathematical modeling of the magnetoactive object's (MO) motion, we consider the computational scheme presented in Figure 1.

In the general case, the motion of a magnetoactive object can be described by the equation (1):

$$\{\begin{array}{c} m\frac{d^{2}q}{dt^{2}}={\vec{F}}_{m}+{\vec{F}}_{d}+{\vec{F}}_{fr}+{\vec{F}}_{e}+{\vec{F}}_{GA}+{\vec{F}}_{c};\\ \left(\frac{2mr^{2}}{5}\right)\frac{d^{2}\phi }{dt^{2}}=M_{m}+{\vec{F}}_{fr}r-M_{fr}, \end{array}$$ (1)

For the planar x−z setting considered in this study, we define the generalized coordinate vector as $q=[x,\;z,\;\;\phi {]}^{⊤}$, where *x*(m) is the axial position of the MO along the channel, *z*(m) is the wall-normal (vertical) position (positive upward), and ϕ(rad) is the in-plane rotation angle of the MO about the out-of-plane *y*-axis. Time derivatives are denoted by overdots: $\dot{q}=[\dot{x},\;\;\dot{z},\;\;\dot{\phi }{]}^{⊤}$ and $\ddot{q}=[\ddot{x},\;\ddot{z},\;\;\ddot{\phi }{]}^{⊤}$. Equation (1) represents the Newton–Euler balance of the magnetoactive object (MO). In expanded form, the translational and rotational balances can be written as: $m\ddot{x}=\Sigma F_{x},m\ddot{z}=\Sigma F_{z},J\ddot{\phi }=\Sigma M_{y},$ where *m* is the MO mass, *r* is the MO radius, and *J* is the mass moment of inertia about the *y*-axis (for a homogeneous sphere $J=2/5\;mr^{2}$). $\Sigma F_{x}$ and $\Sigma F_{z}$ denote the resultant force components acting on the MO in the axial and wall-normal directions, respectively, and $\Sigma M_{y}$ is the resultant moment about the *y*-axis.

Throughout Section 2, these resultants are composed of: the magnetic ponderomotive force $F_{m}\;$ (Section “Magnetic Force and Torque”), viscous drag $F_{d}$ (Section “Drag force and Torque”), dry friction and rolling resistance during wall contact (Section “Friction forces”), viscoelastic wall-contact interaction (Section “Contact force”), and the net gravity–buoyancy force $F_{g}$ (Section “Gravity and buoyant forces”). The normal reaction N≥0 is defined as acting from the wall toward the MO. In the present model, the torsional magnetic moment $M_{m}$ is neglected as justified below; therefore, $\Sigma M_{y}$ is dominated by contact-induced moments and by the kinematic constraints imposed by rolling regimes.

#### 2.1.1 Magnetic force and torque

In this study, a magnetic object (MO) made of a silicone polymer is used, containing randomly oriented micron-sized carbonyl iron particles (no larger than 100 μm) with a final particle content of 40%. Since the magnetic moments of individual ferromagnetic particles within the MO are chaotically oriented, the microrobot possesses no intrinsic magnetic moment. However, under an external magnetic field, the particles can partially align with the field, generating weak magnetization and exhibiting paramagnetic properties.

Thus, the magnetic ponderomotive force *F_m_* arises due to the interaction between the induced magnetic moment in the MO: $\vec{m}=V\chi \vec{H}$ (where *χ* is the magnetic susceptibility and *V* is the volume of the microobject) and the external magnetic field *H* of a permanent magnet. This force can be determined using Equation (2):

$${\bar{F}}_{m}=\mu_{0}(\bar{m}\cdot \nabla )\bar{H},$$ (2)

where: *μ*_0_—vacuum magnetic permeability, ∇–vector differential operator. The magnetic field strength is determined by the vector sum of its components $H=\sqrt{H_{x}^{2}+H_{y}^{2}+H_{z}^{2}}$, which are calculated in this work through the numerical solution of the equations (3):

$$\begin{array}{rl} & H_{x}=-\frac{M}{4\pi }\overset{2}{\underset{i,j,k=1}{\sum }}(-1{)}^{i+j+k}\ln\left(y_{j}+\sqrt{x_{i}^{2}+y_{j}^{2}+z_{k}^{2}}\right);\\ & H_{y}=-\frac{M}{4\pi }\overset{2}{\underset{i,j,k=1}{\sum }}(-1{)}^{i+j+k}\ln\left(x_{i}+\sqrt{x_{i}^{2}+y_{j}^{2}+z_{k}^{2}}\right);\\ & H_{z}=-\frac{M}{4\pi }\overset{2}{\underset{i,j,k=1}{\sum }}(-1{)}^{i+j+k}\mathrm{arctg}\left(\frac{x_{i}y_{j}}{z_{k}\sqrt{x_{i}^{2}+y_{j}^{2}+z_{k}^{2}}}\right), \end{array}$$ (3)

where $x_{i}=x+(-1{)}^{i}a/2$, $y_{j}=y+(-1{)}^{j}b/2$, $z_{k}=z+(-1{)}^{k}c/2$, *M—*the magnetization of the magnet (along the *z* axis), *x*, *y*, *z*—are the displacements of the center of mass of the magnetic object (MO) relative to the center of the permanent magnet; *a, b, c—*are the dimensions of the permanent magnet (an edge length of 20 mm is used in this work).

For clarity, $[H_{x},H_{y},H_{z}{]}^{⊤}$ denotes the magnetic field strength vector H evaluated at the MO center. The relative coordinates (x,y,z) used in Equation (3) are the displacements from the permanent magnet center to the MO center. In the present planar experiments and simulations, the magnet-to-channel offset in the *y*-direction is fixed ($y=y_{0}$ constant), while x and *z* coincide with the generalized coordinates defined after Equation (1).

Note that under significant deformations of the magnetic object (MO), the demagnetizing factor will not be uniform across different directions. This non-uniformity may lead to the emergence of a torsional magnetic moment *M_m_*, which tends to align the MO along the magnetic field isolines. Consequently, this effect could hinder certain modes of motion, such as rolling along the channel wall. However, the results earlier studies (26) justify neglecting this influence, assuming that only the ponderomotive force *F_m_* acts on the MO.

#### 2.1.2 Drag force and torque

An important factor in describing the dynamics of MO motion inside a channel is the viscous drag force. In this work, the fluid is modeled as Newtonian. This simplification is justified by the primary focus on validating the contact-aware control framework: it isolates the effects of the control algorithms from complex rheology and is adequate under the low Reynolds number (Re ≪ 1) and quasi-steady conditions of our experiments, where viscous forces dominate.

The work of Arcese, Fruchard, and Ferreira (21) presents a model accounting for wall-proximity effects, manifested as an increased drag force. This effect is explained by the emergence of excess pressure ahead of the MO, creating an additional resistive force. Applying this model would, in principle, preclude any motion of the MO along the wall under a magnetic force. However, experiments (27) have demonstrated such motion. The aim is to create a phenomenological approximation that qualitatively captures the key trend—a sharp increase in resistance with decreasing gap *δ*—while remaining computationally efficient for integration into a real-time control loop and for conducting multiple parametric simulations. It is critically important that this approximation is consistent with experimental observations regarding the fundamental possibility of motion.

To achieve this, we refine the model by introducing a constraint on the wall-proximity coefficient β₀, based on experimental data, and by accounting for surface irregularities through a minimum gap δ:

$${\vec{F}}_{d}=\frac{\rho_{f}SC_{d}}{2}\left(\frac{\left|\overset{\dot{-}}{\vec{q}}{\vec{\nu }}_{f}\right|}{\beta_{0}+\delta /r}\right)\mathrm{sign}\left(\overset{\dot{-}}{\vec{q}}{\vec{\nu }}_{f}\right).$$ (4)

Here, *ρ_f_* is the fluid (blood) density inside the channel; *β*_0_ is the resistance coefficient during MO contact with the channel surface (governing motion capability during contact); *δ* is the gap size between the MO and the supporting surface; *ν_f_* is the fluid flow velocity; *S* is the frontal area of the microrobot (for a spherical MO, *S* = *πr*^2^); and *C_d_* is the drag coefficient, which depends on the Reynolds number.

#### 2.1.3 Friction forces

The friction force model plays a crucial role in describing the motion regimes of a magnetic object (MO) during contact with channel walls. The combination of friction force and rolling friction torque upon wall contact determines the motion mode: rolling, sliding, or rolling with slipping. In the general case, both surface reactions depend on the normal reaction force: *F_fr_* *=* *k_fr_N*, *F_fr.r_* *=* *k_r_N/r,* where *k_fr_* is the sliding friction coefficient and *k_r_* is the rolling friction coefficient. Thus, the condition for the presence of sliding is determined by the relation: *∑F_x_* *≤* *F_fr_*∧*∑F_x_* *≥* *F_fr.r_*.

Note that the normal reaction force N is determined during the solution of the system of differential equations describing the MO's motion dynamics.

This study employs an original algorithm that enables the implementation of various motion types while accounting for nonlinear effects of MO interaction with the channel surface. Since motion along the wall involves both rotational and translational components, several motion regimes are possible:

1. Stationary state (initiation of motion): *dx*/*dt* = 0, *dϕ*/*dt* = 0.

Here, the friction force is determined from the first equation of system (1) and is approximately equal to the horizontal component of the magnetic force *F_m_*, not exceeding the threshold value required to initiate motion.

2. Sliding without rolling: *dx*/*dt* ≠ 0, *dϕ*/*dt* = 0.

This regime occurs when the sliding friction torque *F_fr_r* is insufficient to overcome the rolling friction torque *M_fr_*, causing the object to slide without rotation. This mode is suitable for describing asymmetrically shaped or highly deformable MO.

3. Pure rolling without slipping: (*dx*/*dt* − *r·dϕ*/*dt*) = 0

In this case, the friction force *F_fr_* is determined from the equation of motion to ensure MO-surface adhesion. Rotational and translational motions are kinematically linked (*dx*/*dt* = *r·dϕ*/*dt*), and the rolling friction torque *M_fr_* reaches its limiting value.

4. Rolling with slipping: (*dx*/*dt* − *r·dϕ*/*dt*) ≠ 0

This is the most general and complex MO motion regime. It requires independent integration of motion equations while monitoring the slip magnitude (*dx*/*dt* − *r·dϕ*/*dt*), which determines the direction of the friction force. Two sub-regimes are possible: positive slip (*dx*/*dt* > *r·dϕ*/*dt*), the center of mass moves faster than in pure rolling, as the MO fails to accelerate rotationally due to friction at the contact point (*F_fr_*). Negative slip (*dx*/*dt* < *r·dϕ*/*dt*): The MO rotates faster than in pure rolling, which may occur during inertial rotation or a sudden decrease in the rolling friction coefficient *k_r_*.

In general, the sliding friction force will be determined according to model by Malchikov, Yatsun, Ryapolov & Sokolov (26):

$$F_{\;fr}=\{\begin{array}{c} -(F_{0}-b_{1}\dot{x}+b_{3}{\dot{x}}^{3})\mathrm{sign}(\dot{x})\mathrm{if}\;(|\dot{x}|>0);\\ -\sum F\mathrm{if}\;(\dot{x}=0)\wedge \left(\left|\sum F\right|<k_{\;fr}N\right);\\ -k_{\;fr}N\mathrm{sign}\left(\sum F\right)\mathrm{if}\;(\dot{x}=0)\wedge \left(\left|\sum F\right|\geq k_{\;fr}N\right), \end{array}$$ (5)

where: *F*_0_—experimentally determined component of dynamic friction (*F*_0_ < *k_fr_N*), *b*_1_, *b*_3_—the coefficients of dynamic friction characteristics that determine the dependence of the amount of dry friction on the speed of movement, *k_fr_*—is the coefficient of static dry friction. The parameters of the nonlinear friction model (*F*₀, *b*₁, *b*₃) were determined experimentally on a specialized test bench by measuring the friction force as a function of velocity under various normal loads corresponding to the system's operational range, followed by data approximation with a cubic polynomial. Here, $\Sigma F_{x}\;$ denotes the resultant axial force component acting on the MO [computed from the balance in Equation (1), excluding the dry-friction term itself], and $v=\dot{x}$ is the MO axial velocity along the wall.

It should be noted that significant MO deformation, which leads to an increased contact area, can also increase the sliding friction coefficient due to additional adhesion effects. However, this influence is considered secondary in the context of this study. The primary effect of an increased contact area under load, namely the associated increase in rolling resistance, is accounted for in the model through the variable rolling friction coefficient, *k_r_*, as shown in Equation (6).

The rolling friction torque can be described by the following system of equations:

$$M_{\;fr}=\{\begin{array}{c} -(k_{r0}+\gamma N)N\cdot \mathrm{sign}\left(\sum F\right)\mathrm{if}\;(|\dot{\phi }|>0);\\ -(k_{r0}+\gamma N)N\cdot \mathrm{sign}(\dot{\phi })\mathrm{if}\;(|\dot{\phi }|\leq 0). \end{array}$$ (6)

The use of a nonlinear rolling friction coefficient allows accounting for MO deformation under significant vertical force components. Indeed, for magnetoactive objects made of magnetic particle-filled silicone polymer, substantial deformation is observed at high values of the vertical component of magnetic force. This deformation increases the contact area between the MO and the surface, consequently increasing rolling resistance.

In the proposed model (6), this effect can be accounted for by introducing a variable rolling friction coefficient: *k_r_* = *k_r0_* *+* *γN*, where *k_r0_* represents rolling friction under small loads (determined by material stiffness), *γ* is an experimentally determined parameter.

#### 2.1.4 Electrostatic force

The electrostatic force between the microrobot and the channel wall can be described by the equation (28):

$${\vec{F}}_{e}=\frac{q_{e}^{2}}{4\pi \varepsilon \varepsilon_{0}}\left(\frac{1}{{\left(r+\delta \right)}^{2}}\right),$$ (7)

where: *q_e_* is the microrobot's inherent charge; *ε*_0_ is the vacuum permittivity; *ε* is the relative permittivity of the medium inside the channel; *δ* is the distance between the robot surface and the vessel wall.

This model assumes that the vessel walls are electrically neutral and that the force arises solely from induced charges generated when an electrically charged MO passes through them. However, this study employs an MO with no intrinsic electric charge, while triboelectrically induced charges from fluid friction remain negligible. An order-of-magnitude estimation for our experimental conditions (dielectric materials in a conductive aqueous solution) shows that electrostatic forces (∼10^−^^12^–10^−^^13^ *N*) are 3–4 orders of magnitude smaller than the dominant magnetic (∼10^−^^6^ N) and hydrodynamic (∼10^−^^7^ N) forces. Therefore, within the scope of this benchtop model, electrostatic interactions are excluded from the modeling framework. We note that this simplification may need to be revisited for modeling in biological environments (e.g., blood with ionic composition and charged cellular components).

#### 2.1.5 Gravity and buoyant forces

The magnetic particle-filled silicone sphere investigated in this study is subject to gravitational and buoyant forces. The resultant net force can be described by the equation:

$${\vec{F}}_{GA}=V(\rho -\rho_{f})g,$$ (8)

where: *ρ* is the average density of the robot (composite of magnetic material and polymer); *ρ_f_* is the fluid density.

The magnetoactive object used in this work has a density significantly higher than that of the surrounding fluid. Consequently, the MO will tend to sediment toward the channel's bottom wall.

#### 2.1.6 Contact force

An essential aspect of this work involves modeling both the channel geometry and contact interactions between the MO and channel walls.

A common practice in modeling the interaction between solid bodies and vessel walls is the use of the elastic contact model (Hertzian model). This approach effectively captures the elastic properties and mutual deformation of interacting objects (29, 30). However, these models do not take into account energy dissipation during interaction. Therefore, this study uses a viscoelastic model (the Kelvin-Voigt model), which combines both elastic and viscous behavior:

$${\vec{F}}_{c}=\{\begin{array}{c} 0ifq_{n}<q_{w}\\ c_{w}(q_{w}-q_{n})-\mu_{w}\dot{q} \end{array}$$ (9)

where $q_{n}\;$ denotes the MO coordinate along the contact normal (in this work, the wall-normal/vertical direction), $q_{w\;}$ is the corresponding wall position given by the channel model (for the present linear-wall approximation, $q_{w}\equiv z_{w}\;$ is constant), and $c_{w}\;$ and $\mu_{w\;}$ are the effective contact stiffness and damping coefficients of the Kelvin–Voigt model.

When modeling the interaction, the vessel boundary can be mathematically described using a functional representation (e.g., harmonic). However, to ensure computational experiment reproducibility, this work approximates the wall as a linear boundary with constant z-axis position.

### 2.2 Mathematical model of an electromechanical permanent magnet linear actuator system

Let's consider the scheme of an electromechanical permanent magnet drive system with three degrees of freedom This configuration generates a controlled spatial gradient of the magnetic field in the vicinity of the MO, enabling precise manipulation of its position and orientation.

**Figure 2:**
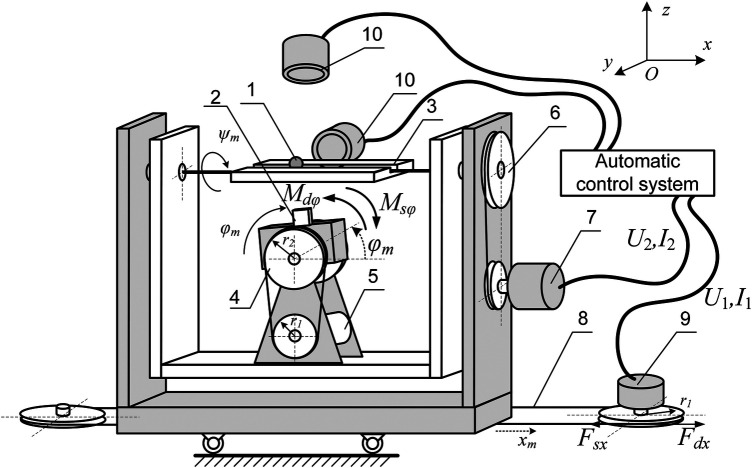
Schematic of the untethered benchtop testbed used to evaluate the proposed contact-aware control framework. The setup includes permanent-magnet actuation, orthogonal vision tracking, and real-time control computation.

The schematic (Figure 2) illustrates the following key components: 1—мagnetoactive object (MO) (the target of magnetic manipulation); 2—cubic permanent magnet (field source for MO actuation); 3—sealed microchannel (confinement structure for the MO moving); 4, 5—*ϕ*-axis rotation drive(transmission + servomotor); 6, 7—*ψ*-axis rotation drive (transmission + servomotor); 8, 9—*x*-axis linear drive (transmission + servomotor), 10—optical tracking cameras.

The actuation system utilizes synchronous servo motors equipped with integrated optical encoders for precise motion control. The permanent magnet is rigidly mounted on the *ϕ_m_* rotational axis, with the MO's path aligned collinearly with the *ψ_m_* axis. The experimental setup maintains a fixed magnet-to-MO separation distance within the *yOz* plane. Through coordinated magnet rotation in both *yOz* and *xOz* planes coupled with translational displacement along *x_m_*, the system achieves independent control over both the directionality and intensity of the applied magnetic force vector.

Note that magnet rotation around the MO's trajectory is used to navigate channel bifurcations, as described in (31). This study focuses exclusively on horizontal displacement and magnet rotation about the *Oy* axis, which modulates the ratio between vertical and horizontal magnetic force components to achieve different MO motion regimes.

A computer vision system, comprising two orthogonally aligned cameras (oriented along *Oy* and *Oz* axes), provides real-time tracking of the MO within the transparent polyacrylate channel. This dual-camera arrangement significantly improves spatial resolution and tracking reliability compared to single-camera systems.

To describe the dynamics of an electromechanical system for moving a magnet, we will use the equations:

$$\left\{ \begin{array}{c} {\ddot{x}}_{m}(m_{1}+m_{2})=F_{dx}-F_{sx};\;\\ F_{dx}=\left(\frac{k_{m1}I_{1}}{r_{1}}\right)\eta_{x};\;\\ {\dot{I}}_{1}=\left(U_{1}-R_{a1}I_{1}-\frac{k_{e1}}{r_{1}}{\dot{x}}_{m}\right)/L_{a1};\;\\ {\ddot{\phi }}_{m}\left(\frac{m_{2}a^{2}}{6}\right)=M_{d\phi }-M_{s\phi };\;\\ M_{d\phi }=\left(\frac{k_{m2}I_{2}r_{2}}{r_{1}}\right)\eta_{\phi };\;\\ {\dot{I}}_{2}=\left(U_{2}-R_{a2}I_{2}-\frac{k_{e2}r_{2}}{r_{1}}{\dot{\phi }}_{m}\right)/L_{a2}.\; \end{array}\right.$$ (10)

The equation (10) includes the following parameters of the electric drives: *k_m_*_1_ and *k_m_*_2_ represent the motor torque constants, *k_e_*_1_ and *k_e_*_2_ denote the motor velocity constants, *η_x_* and *η_φ_* indicate the efficiency coefficients for linear and rotary drives respectively, *R_a_*_1_ and *R_a_*_2_ correspond to the armature winding resistances of the motors, while *L_a_*_1_ and *L_a_*_2_ represent the armature winding inductances.

The control inputs are the motor voltages $U_{1}$ and $U_{2}$, generated by the corresponding control regulators.

### 2.3 Contactless control system

We developed an untethered benchtop testbed to experimentally evaluate the proposed contact-aware control strategy under controlled and repeatable conditions. The platform comprises (i) a permanent-magnet actuation stage, (ii) orthogonal vision-based tracking, and (iii) a real-time control loop that computes the commanded actuation based on the measured MO state. Throughout the manuscript, “contact-aware” refers to explicitly modeling and regulating wall contact forces within the controller, whereas “untethered” describes the experimental setup in which the MO is not mechanically connected to external components.

In this article, movement along the channel surface is considered, in which case the control system provides only the required horizontal movement x*. In the future, it is planned to expand the functionality of the control system for monitoring and vertical movement.

**Figure 3:**
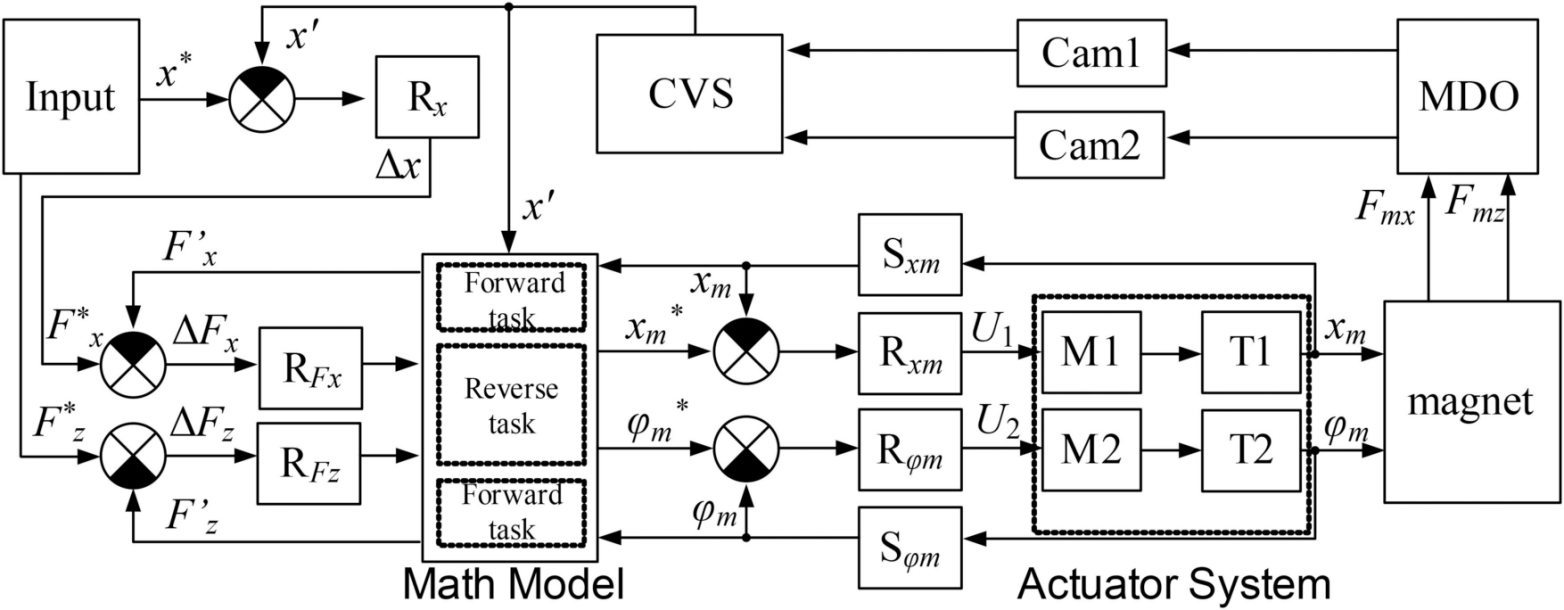
Block diagram of the contact-aware control architecture. The outer loop regulates position tracking, while the inner loop uses model-based force estimation to regulate wall-contact load via the vertical magnetic-force component.

**Figure 4:**
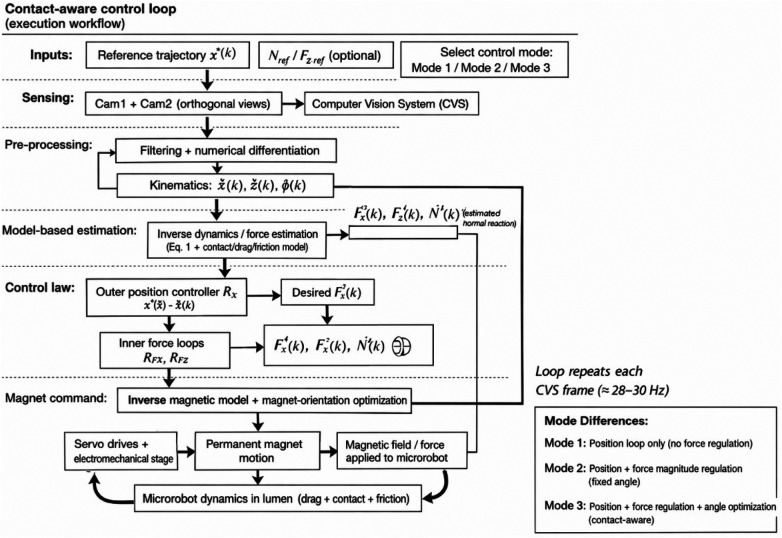
Step-by-step execution flowchart of the proposed method at each vision update (≈28–30 Hz): image acquisition and state reconstruction, velocity estimation, model-based inverse-dynamics force inference, control-law evaluation for the selected mode, inverse magnetic mapping with magnet-orientation optimization, and actuation of the permanent magnet.

End-to-end workflow of the proposed method. While the functional architecture is shown in Figure 3, an explicit execution workflow is provided in Figure 4 to clarify how sensing, model-based estimation, and control actions interact at each update step. In brief, the system acquires synchronized images from two orthogonal cameras, reconstructs the microrobot pose, estimates kinematics and interaction forces through inverse dynamics, and then computes the magnet position and orientation required to realize the desired magnetic force components under the selected control mode. This closed-loop sequence is executed at the effective computer-vision update rate (≈28–30 Hz), and repeats continuously along the reference trajectory, thereby making the proposed approach reproducible as a step-by-step algorithm rather than only a block-level concept.

The implemented control architecture employs a dual-camera vision system (Cam1, Cam2) for real-time monitoring of the MO's positional coordinates (*x*′) within the channel. The acquired visual data undergoes processing through a dedicated computer vision system (CVS). The control sequence initiates with the comparison between a reference displacement value (x*), provided via input device, and the actual MO position, thereby generating a positional error signal. This error term serves as input to the R*_x_* controller, which computes the requisite horizontal magnetic force component (F*_x_).

Subsequent to this computation, the system performs a comparative analysis between *F_x_* and the calculated force value (*F'_x_*), with the resultant error term being processed by the R*_Fx_* controller before transmission to the mathematical modeling block. An analogous control pathway exists for the vertical force component, where the reference value (*F_z_*) is continuously compared with its calculated counterpart (*F'_z_*), with the error term being processed by the R*_Fz_* controller prior to model input.

The mathematical model block executes two principal computational functions:

Solution of the direct problem—determination of ponderomotive force (*F_m_*) projections based on known spatial configurations of both magnet and MO
Solution of the inverse problem—computation of required magnet position and orientation parameters to achieve specified force projections (*F_m_*) given the MO's current position

The direct problem solution is achieved through numerical implementation of the magnetic force equations (2), (3). The inverse problem presents greater computational complexity, requiring iterative exploration of the magnet's parametric space to identify configurations yielding the desired output parameters. This inverse solution process involves systematic numerical probing of the model's parameter space to derive optimal magnet positioning parameters.

Based on the measured positions *x'*(*k*), *ϕ'*(*k*) (where *k—*is the discrete-time index) and their derivatives, as well as the current force estimates, the mathematical model block dynamically solves an inverse dynamics problem. It determines the forces (*F'_m_*, *N*′) which, when applied to the model (Equation 1), would produce the observed motion of the MO. Thus, the model continuously refines the estimates of *F'_x_*, *F'_z_*, and the normal reaction *N*′ used in the R*_Fx_* and R*_Fz_* control loops. This algorithm operates at the CVS update rate of 28–30 Hz.

To enable contact-aware regulation without direct force sensing, the control architecture includes a model-based force estimation loop driven by vision measurements (Figure 3). The mathematical model block uses the measured object pose, namely the longitudinal position and orientation $x^{\prime }(k)$ and $\phi^{\prime }(k)$, sampled at discrete time, *k* and computes their time derivatives by numerical differentiation (with smoothing to reduce amplification of measurement noise). The resulting kinematic estimates, together with the dynamic model of the magnetoactive object (Equation 1) and the current model parameters, are used to solve an inverse-dynamics problem at each update step.

Specifically, the estimator determines the magnetic force projections and contact load that best explain the observed motion over the most recent interval. In our notation, this produces updated estimates of the horizontal and vertical magnetic force components $\left(F_{x}^{\prime },F_{z}^{\prime }\right)$ and the corresponding normal reaction *N*′. These estimates are then used in the inner force-related loops (RFx and RFz) as a predictive compensation term rather than as a standalone force controller: their role is to improve disturbance rejection (e.g., due to drag/friction variability) while the outer position loop (Rx) guarantees tracking stability.

The force estimation loop runs at the effective update rate of the computer vision system (CVS), which is limited by image acquisition and processing and is approximately 28–30 Hz in our setup. Consequently, force updates are discrete-time and may exhibit small latency relative to the motor drive dynamics; this is accounted for by treating the estimates as feedforward corrections rather than hard force-tracking commands.

Because $F_{x}^{\prime }$, $F_{z}^{\prime }$ and *N*′ are inferred from kinematics and a simplified dynamic model, estimation accuracy is primarily affected by (i) CVS measurement noise and calibration errors, (ii) numerical differentiation of $x^{\prime }(k)$ and $\phi^{\prime }(k)$, (iii) model mismatch in drag/friction/contact regime switching, and (iv) uncertainty in the magnetic force mapping used in the direct model. Importantly, such errors do not destabilize the system because the outer-loop position feedback corrects residual tracking errors; the practical impact is limited to reduced feedforward effectiveness and slightly larger transient tracking deviations that are subsequently compensated by Rx.

The reference position and angular values generated by the mathematical model undergo continuous comparison with feedback signals from position sensors (S*_xm_*, S*_φm_*). The resulting error signals are processed by drive controllers (R*_xm_*, R*_φm_*), which generate appropriate motor excitation voltages. These control signals actuate motors (M1, M2), which in turn effect precise magnet displacement through mechanical transmission systems (T1, T2).

It is important to note that the force estimation loop acts not as a standalone force controller, but as a predictive compensator (feedforward). Its purpose is to improve the system's disturbance rejection, not to achieve perfect force tracking. The ultimate positioning accuracy is guaranteed by the primary outer position feedback loop (R*_x_*). Therefore, inevitable force estimation errors do not cause instability but only result in a suboptimal feedforward action, manifesting as a slightly larger positional error that is subsequently corrected by the R*_x_* controller.

Next, the changed magnetic field sets the MO in motion, which is recorded with the help of cameras and the cycle of the control system repeats.

### 2.4 Experimental setup

**Figure 5:**
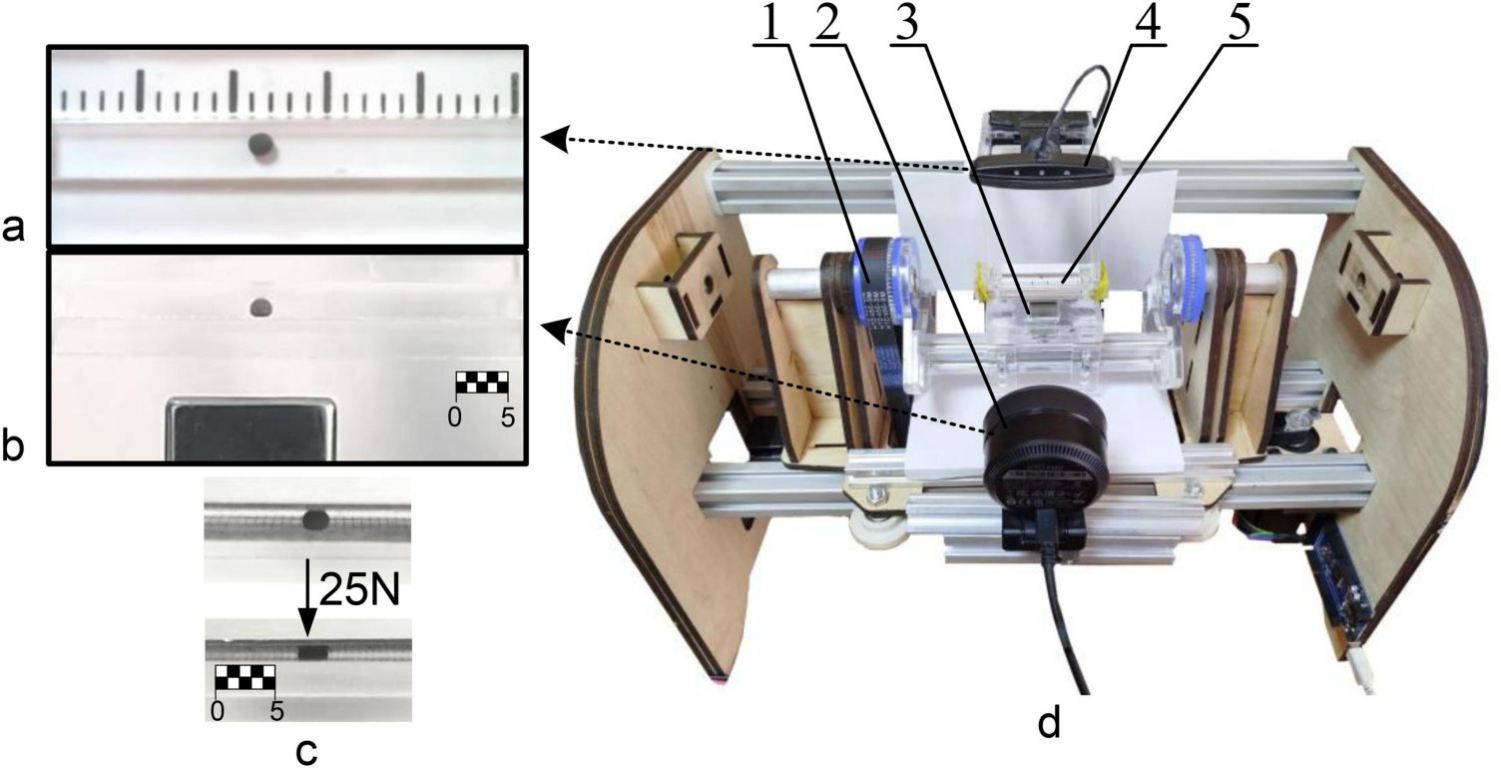
Experimental platform and MO visualization. **(a)** Overhead camera view of the MO in the channel. **(b)** Side camera view used for vertical state estimation. **(c)** Representative MO deformation under normal load. **(d)** Photograph of the benchtop setup: (1) drive stage, (2) side-view camera, (3) permanent magnet, (4) top-view camera, and (5) channel containing the MO.

To clarify the unknown parameters of the model and verify the results of numerical simulation, a set of experimental studies was performed on a laboratory bench (25), shown in Figure 5d.

The test bench configuration fundamentally consists of an electromechanical drive system for magnet positioning, a machine vision system for MO motion tracking within the channel, and the microchannel assembly housing the MO. Comprehensive details regarding the bench construction, control system architecture, as well as the computer vision and automatic control algorithms are provided in the authors' previous publication (25). The current investigation particularly focuses on monitoring MO rotation during motion, accomplished through dual orthogonal camera tracking (90 ° configuration) of high-contrast markers applied to the MO surface.

A significant portion of this work involves the development of deformable spherical magnetoactive objects. Figure 4 presents an MO specimen with 1.8 mm diameter, demonstrating its deformed state under 25 N compressive load. The fabrication process employed a two-component silicone compound selected for its high initial fluidity, which upon curing yields an elastomeric matrix exhibiting substantial deformability and enhanced frictional properties.

Paramagnetic characteristics were imparted through incorporation of carbonyl iron particles (≤100 μm) at 40% volumetric concentration. The manufacturing protocol involved precision mold fabrication using photopolymer 3D printing followed by casting of the magnetoactive composite. Identification markers were applied using thin acrylic paint layers, carefully controlled to minimize geometric interference.

It should be noted that the surface properties of the acrylic markers significantly influence MO dynamics during experimental trials. Therefore, only those experimental segments where markers remained out of contact with the channel surface were considered for analysis, ensuring unaffected research outcomes.

### 2.5 Simulation protocol and robustness analysis

To strengthen the reproducibility and robustness assessment of the proposed control framework, we performed a simulation-based Monte Carlo analysis using the same mathematical model and controller structure reported in this manuscript. Simulations were run in discrete time with a fixed step size and a sinusoidal reference trajectory along the channel axis. The reference was defined as $x^{\setminus *}(t)=A_{m}\sin(2\pi ft)$, where *A_m_* and *f* are the reference amplitude and frequency, respectively. The simulation horizon was *T* seconds, starting from the initial condition $x(0)=x_{0}$. The robustness analysis was conducted independently for each control mode (mode 1–3), keeping controller gains unchanged and perturbing only selected uncertain plant parameters and measurement noise, as described below.

We implemented a Monte Carlo protocol with n=500 trials per control mode and a fixed random seed to ensure exact reproducibility of the reported distributions. In each trial, we perturbed a subset of parameters that (i) directly influence wall–contact loading and tracking performance, and (ii) are expected to vary across experiments and future translational environments. Specifically, we applied multiplicative Gaussian perturbations to the drag coefficient $\mu_{F}$, the dynamic sliding friction coefficient $k_{\;frD}$, and the electromechanical coupling parameter couple_c, according to $\tilde{\;p}=p(1+\varepsilon )$, with $\varepsilon ∼N(0,\sigma_{p})$. In addition, we injected additive zero-mean Gaussian measurement noise in the observed position x(t), with standard deviation $\sigma_{x}$, to emulate computer-vision tracking uncertainty. All Monte Carlo settings and uncertainty levels are summarized in Table 1.

**Table 1:** Monte carlo settings and uncertainty model used for simulation robustness analysis.

Category	Parameter (symbol)	Nominal value	Uncertainty/distribution (Monte Carlo)
Time discretization	Time step (dt)	0.01 s	Fixed (no perturbation)
Simulation horizon	Duration (T)	12.0 s	Fixed (no perturbation)
Reference trajectory	Amplitude (A_m)	0.018 m	Fixed (no perturbation)
Reference trajectory	Frequency (f)	0.25 Hz	Fixed (no perturbation)
Initial condition	Initial position (x0)	0.0 m	Fixed (no perturbation)
Monte Carlo protocol	Trials per mode (*n*)	500	Fixed
Monte Carlo protocol	Random seed	7	Fixed (reproducibility)
Measurement model	Position noise (*σ*_x)	5 × 10^−^^5^ m	Additive Gaussian: $x_{meas}=x+\eta$, $\eta ∼N(0,\sigma_{x}^{2})$
Hydrodynamic parameter	Drag coefficient (μ_F)	1 × 10^−8^	Multiplicative Gaussian: ${\tilde{\mu }}_{F}=\mu_{F}(1+\varepsilon )$, ε∼N(0,0.15)
Contact parameter	Dynamic sliding friction (k_frD)	0.15	Multiplicative Gaussian: ${\tilde{k}}_{\;frD}=k_{\;frD}(1+\varepsilon )$, ε∼N(0,0.15)
Electromechanical coupling	Coupling parameter (couple_c)	0.3	Multiplicative Gaussian: $\tilde{couple\_c}=couple\_c(1+\varepsilon )$, ε∼N(0,0.20)
Safety/constraints	Force limit (Fmax)	6 × 10^−6^	Fixed (hard limit)
Safety/constraints	Normal reaction bounds (N_min, N_max)	0, 3 × 10^−^⁶	Fixed (hard limits)

For every trial we computed the same performance metrics used throughout the manuscript: (i) the RMS tracking error $E_{r}$, (ii) the maximum absolute tracking error $E_{rMax}$, (iii) the normal-load index $I_{n}$, and (iv) the peak normal reaction $N_{Max}$. Here, $E_{r}=\sqrt{1/T\int_{0}^{T}{\left(x(t)-x^{\setminus *}(t)\right)}^{2}dt}$, $E_{r\mathrm{Max}}=\mathrm{ma}x_{t\in [0,T]}|x(t)-x^{\setminus *}(t)|$, $I_{n}=\int_{0}^{T}N{\left(t\right)}^{2}dt$, and $N_{\mathrm{Max}}=\mathrm{ma}x_{t\in [0,T]}N(t)$. The resulting distributions (mean and standard deviation across trials) were then used to characterize robustness and between-mode separability under parametric uncertainty and measurement noise.

### 2.6 Statistical analysis

All statistical tests reported in this study are simulation-based, derived from the Monte Carlo robustness evaluation described above (*n* = 500 trials per control mode). Because the metric distributions are not assumed to be Gaussian and may exhibit heteroscedasticity under parameter perturbations, we used non-parametric hypothesis tests to compare control modes. For each performance metric ( $E_{r}$, $E_{r\mathrm{Max}}$, $I_{n}$, and $N_{\mathrm{Max}}$), we first performed a global Kruskal–Wallis test across the three control modes to assess whether at least one mode differs from the others. When the global test was significant, we conducted pairwise two-sided Mann–Whitney U tests for all mode pairs.

To control the family-wise error rate due to multiple pairwise comparisons, we applied the Holm correction to the pairwise *p*-values (reported as $p_{\mathrm{Holm}}$). In addition to statistical significance, we quantified the magnitude of between-mode differences using Cliff's delta (δ), a distribution-free effect size that reflects the degree of stochastic superiority between two samples (|δ| close to 1 indicates near-complete separation; |δ| near 0 indicates substantial overlap). The complete set of global *p*-values, Holm-corrected pairwise *p*-values, and effect sizes for each metric is provided in Table 2.

**Table 2:** Simulation-based statistical testing across control modes (*n* = 500 monte carlo trials per mode).

Metric	Global test (Kruskal–Wallis) *p*-value	Pairwise comparison (Mann–Whitney U)	Holm-corrected *p*-value (pHolm)	Cliff's delta (*δ*)
Er (mm)	1.906e-272	mode1 vs. mode2	6.018e-143	0.930
Er (mm)	1.906e-272	mode1 vs. mode3	3.051e-164	0.999
Er (mm)	1.906e-272	mode2 vs. mode3	4.744e-148	0.948
ErMax (mm)	1.330e-221	mode1 vs. mode2	3.089e-153	0.964
ErMax (mm)	1.330e-221	mode1 vs. mode3	3.369e-154	0.968
ErMax (mm)	1.330e-221	mode2 vs. mode3	6.804e-43	0.502
In (N²·s)	4.605e-290	mode1 vs. mode2	1.757e-164	1.000
In (N²·s)	4.605e-290	mode1 vs. mode3	1.171e-164	1.000
In (*N*²·s)	4.605e-290	mode2 vs. mode3	5.856e-165	1.000
NMax (*N*)	7.127e-289	mode1 vs. mode2	1.757e-164	1.000
NMax (*N*)	7.127e-289	mode1 vs. mode3	1.289e-164	1.000
NMax (*N*)	7.127e-289	mode2 vs. mode3	2.311e-162	0.992

## 3 Results

To evaluate the effectiveness of the proposed automatic control system and verify the results, a set of computational and field experiments were performed. A harmonic law of the following form was chosen as the defining trajectory for the MO movement inside the channel: x* = *A_m_*·sin(*wt*). The different amplitudes in simulation (*A_m_* = −0.03 m, *w* = 1.26 s^−^¹) and experiment (*A_m_*· = −0.018 m, *w* = 0.62 s^−^¹) were due to the technical stroke limitations of the testbed's linear actuator. Unknown system parameters, such as friction coefficients and viscosity parameters, were determined during the study. The main parameters of the mathematical model are presented in Table 3.

**Table 3:** The mathematical model parameters.

Parameter name and designation	Value
Radius of the MO, *r*	0.001 m
Mass of the MO, *m*	1.05 × 10^−8^ kg
Volume of the MO, *V*	4.19 × 10^−9^ m^3^
Magnetic susceptibility, *χ* (for ferromagnetic particle concentration 40%)	0.1
Size of the magnet, *a, b, c*	20, 20, 20 mm
Magnetization directed along z axis, *M*	0.6 × 10^5^ A/m
Viscous drag coefficient, *μ_F_*	10^−8^ kg/s
Static friction coefficient, *k_frS_*	0.25
Sliding friction coefficient, *k_frD_*	0.15
Rolling friction coefficient, *k_r_*_0_	1.1 × 10^−4^ m
Rolling friction model parameter, *γ* (the value was selected numerically)	5.1 × 10^−5^ m/N
Linear motor torque constants, *k_m_*_1_	0.21 Nm/A
Angular motor torque constants, *k_m_*_2_	0.1 6 Nm/A
Linear motor velocity constants, *k_e_*_1_	0.20 Vs
Angular motor velocity constants, *k_e_*_2_	0.15 Vs
Reductors efficiency, *η_x_ η_φ_*	90%
Linear motor winding inductance, *L_a_*_1_	6.8 mH
Angular motor winding inductance, *L_a_*_2_	4.5 mH
Linear motor winding resistances, *R_a_*_1_	1.2 Ohm
Angular motor winding resistances, *R_a_*_2_	0.8 Ohm

To validate the adequacy of the mathematical model and the proposed control system architecture, performance characteristics were obtained for three distinct operational modes.

In the first operational mode, the servo drive control signal was generated solely based on position error (x* − *x*’). In this configuration, the magnet positioning electromechanical system attempted error compensation without considering magnetic force magnitude.

The second operational mode implemented position control to maintain optimal horizontal magnetic force component (*F_mx_*), while lacking active magnet orientation control.

The third mode employed the comprehensive strategy described in preceding sections, where the electromechanical drive system—utilizing data from the mathematical model block—simultaneously optimized both magnet position and orientation.

**Figure 6:**
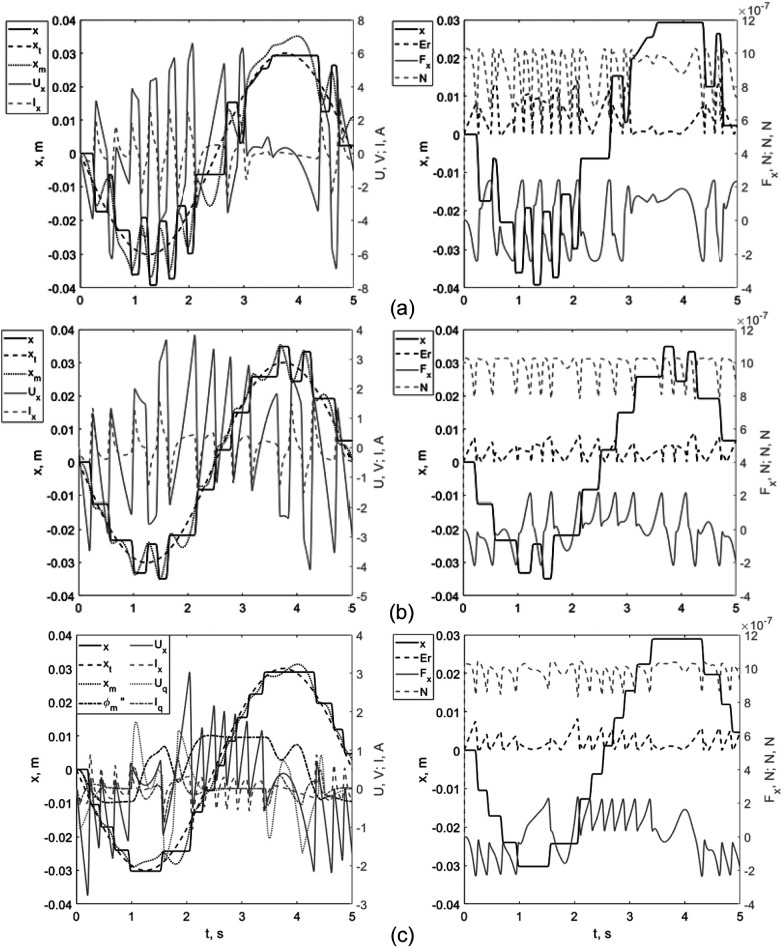
Numerical simulation of MO motion under the three control modes: **(a)** mode 1 (no force regulation), **(b)** mode 2 (force regulation), and **(c)** mode 3 (force-plus-angle regulation). Each panel reports the tracking response relative to the reference trajectory.

The simulation results are presented as time-domain plots in Figure 6.

Control performance was quantified using four complementary metrics: (i) the RMS tracking error $E_{r}$ (root-mean-square deviation between x(t) and $x^{\setminus *}(t)$); (ii) the peak tracking error $E_{r,\max}$; (iii) the normal-load index $I_{n}=\int_{0}^{T}N{\left(t\right)}^{2}\;dt$, which summarizes the time-accumulated contact load during motion; and (iv) the peak normal reaction force $N_{\max}$. Together, these metrics characterize both navigation accuracy and wall-interaction safety.

In the course of the study, each of the three compared control modes was tested in a series of several dozen experiments to ensure statistical reliability. For clarity and conciseness of presentation, the paper shows representative (characteristic) motion trajectories and averaged data for the key metrics presented in Table 4.

**Table 4:** Simulation baseline.

Control quality criteria	Without force control (*φ_m_* = 0)	With force control (*φ_m_* = 0)	With force and angle control
Er, mm	4.8	3.3	2.1
ErMax, mm	14.6	8.8	8
In	4.4 × 10^−10^	3.7 × 10^−10^	1.6 × 10^−10^
NMax	2.0 × 10^−6^	1.9 × 10^−6^	0.8 × 10^−6^

Quantitative robustness and statistical significance are established using a simulation-based Monte Carlo campaign with *n* = 500 realizations per control mode, generated by perturbing the dominant uncertain parameters according to Table 1. Accordingly, the hypothesis testing reported in Table 2 and the metric distributions (and the mean ± SD) are simulation-based and quantify controller robustness under structured model uncertainty. Benchtop experiments are presented as a qualitative validation of the model and closed-loop behavior: we report one representative trajectory-tracking run per mode (total n_bench = 3) to illustrate time-series agreement and relative mode-to-mode improvement. Because full per-trial raw logs from the broader tuning/calibration runs were not preserved in a form that enables recomputation of all metrics (Er, ErMax, In, and NMax) across repeated trials, we avoid inferential claims from the benchtop dataset and base statistical conclusions on the Monte Carlo dataset.

The simulation outcomes unequivocally confirm that incorporating a supplementary force regulation loop (Figure 6b)—wherein the computational model block calculates optimal magnet positioning while compensating for magnetic field inhomogeneities—yields substantial enhancements in system performance. This advanced control strategy, featuring explicit regulation of the horizontal magnetic force component (*F_mx_*), achieves a 31% reduction in positioning inaccuracy (with RMS error diminishing from 4.8 mm to 3.3 mm) and lowers peak displacement from 14.6 mm to 8.8 mm. Furthermore, this methodology produces a 16% decrease in normal contact forces, directly influencing both wall interaction dynamics and magnetoactive object deformation characteristics.

Implementation of magnet orientation control permits optimization of the magnetic force vector through spatial positioning adjustments. Nevertheless, electromechanical system response delays and discontinuous MO motion induced by frictional effects impose fundamental limitations on achievable precision. Experimental validation of the rotational control scheme (Figure 6c) demonstrates an additional 36% improvement in positioning accuracy (final RMS error: 2.1 mm), approaching the physical scale of the MO itself. The most pronounced benefit emerges in normal force mitigation, with the proposed control architecture achieving a 57% reduction and maintaining peak values below 8 × 10^−^⁷ N.

A key aspect of the proposed architecture is that controlling the magnet orientation to reduce normal force simultaneously improves the object's controllability. This mitigates a hard trade-off between accuracy and safety in this problem formulation. During tuning, the normal force acted as a constraint rather than a separate term in the objective function, aligning with the clinical logic of ensuring a safe interaction threshold.

**Figure 7:**
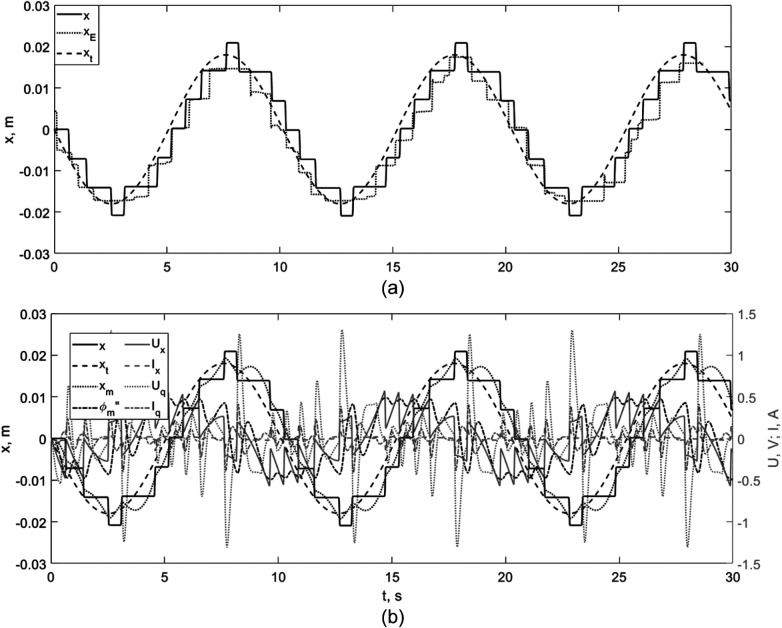
Qualitative agreement between simulation and benchtop experiments. **(a)** Time-series comparison of the axial position x(t) obtained in simulation and experiment. **(b)** Representative dynamic and actuation signals during trajectory tracking, illustrating consistent closed-loop behavior across domains.

Next, we will show the combined graphs of MO movement inside the channel obtained during numerical and field experiments (Figure 7).

Comparative analysis of simulation results (*x* in Figure 7a) and experimental data (*x_E_* in Figure 7a) demonstrates that both the nature and quantitative characteristics of MO movement under the influence of a moving magnet (*x_m_* in Figure 7b) are the same. Optimization of sliding and rolling friction parameters leads to high convergence between the results, confirming the adequacy of the mathematical model developed and validating the algorithms proposed for calculating model parameters for implementing a contactless automatic control system for magnetically active micro-objects using an external magnetic field.

The discrepancy between the experimental and simulation results can be explained by the heterogeneous properties of the channel surface and MO used in the laboratory bench as well as errors in the selection of parameters.

### 3.1 Simulation-based robustness and statistical significance

**Figure 8:**
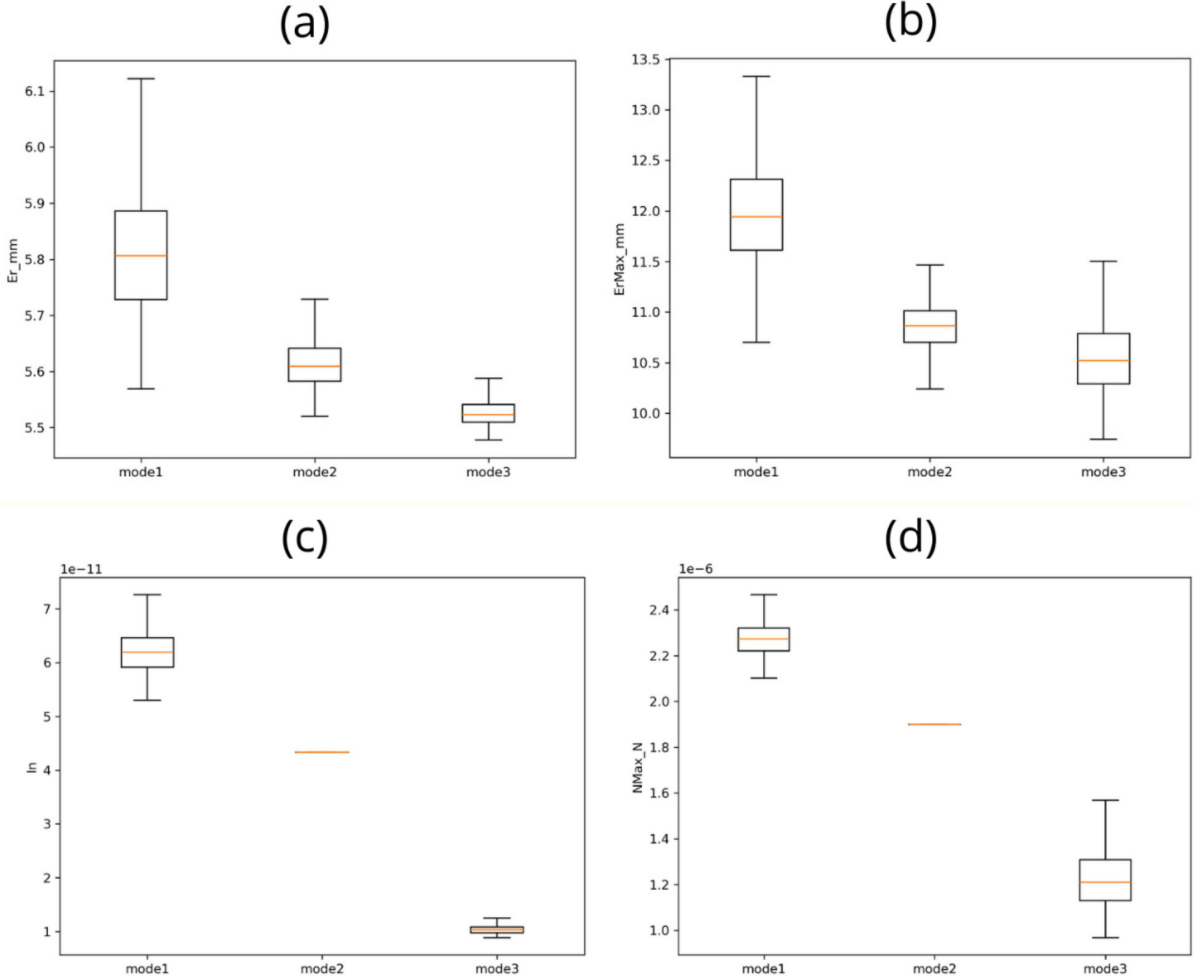
Monte Carlo robustness distributions (*n* = 500 trials per control mode) for tracking and contact-load metrics. Boxplots compare **(a)** RMS tracking error $E_{r}$, **(b)** peak tracking error $E_{r\mathrm{Max}}$, **(c)** normal-load index $I_{n}=\int_{0}^{T}N{\left(t\right)}^{2}dt$, and **(d)** peak normal reaction $N_{\mathrm{Max}}$ across the three control modes under parametric uncertainty and measurement noise. Boxes indicate the interquartile range with the median shown as a horizontal line; whiskers represent the non-outlier range. Contact-aware modes (2–3) reduce contact-load metrics while maintaining or improving tracking performance relative to mode 1.

To further quantify robustness and statistical support of the reported improvements, we performed a simulation-based Monte Carlo analysis (*n* = 500 trials per control mode) using the uncertainty model and protocol described in the Methods section. The resulting distributions of tracking and contact-load metrics are summarized in Figure 8 (boxplots) and Table 5 (mean ± SD across trials). In all cases, the contact-aware strategies (modes 2–3) preserved or improved tracking while reducing contact-load measures compared to mode 1.

**Table 5:** Monte carlo summary statistics (mean ± SD across 500 trials) for simulation robustness analysis.

Control mode	Er (mm), mean ± SD	ErMax (mm), mean ± SD	In (*N*²·s), mean ± SD	NMax (*N*), mean ± SD
mode1	5.814 ± 0.118	11.964 ± 0.500	(6.237 ± 0.435) × 10^−^¹¹	(2.279 ± 0.079) × 10^−^⁶
mode2	5.614 ± 0.043	10.856 ± 0.243	(4.332 ± 0.000) × 10^−^¹¹	(1.900 ± 0.000) × 10^−^⁶
mode3	5.526 ± 0.024	10.570 ± 0.393	(1.043 ± 0.094) × 10^−^¹¹	(1.233 ± 0.156) × 10^−^⁶

Across the 500 perturbed trials, mode 2 reduced the RMS tracking error $E_{r}$ from 5.814±0.118mm (mode 1) to 5.614±0.043mm (−3.45%), and reduced the peak error $E_{r\mathrm{Max}}$from 11.964±0.500mm to 10.856±0.243mm (−9.26%). The full force-plus-angle strategy (mode 3) yielded the best overall performance, reducing $E_{r}$ to 5.526±0.024mm (−4.96%) and $E_{r\mathrm{Max}}$ to 10.570±0.393mm (−11.66%). The reduction in contact-load metrics was more pronounced: the normal-load index $I_{n}$ decreased from $(6.237\pm 0.435)\times 10^{-11}N^{2}s$ (mode 1) to $(4.332\pm 0.000)\times 10^{-11}N^{2}s$ in mode 2 (−30.54%) and to $(1.043\pm 0.094)\times 10^{-11}N^{2}s$ in mode 3 (−83.27%). Likewise, the peak normal reaction $N_{\mathrm{Max}}$ decreased from $(2.279\pm 0.079)\times 10^{-6}N$ (mode 1) to $(1.900\pm 0.000)\times 10^{-6}N$ (mode 2; −16.65%) and to $(1.233\pm 0.156)\times 10^{-6}N$ (mode 3; −45.91%). As illustrated in Figure 8c,d, mode 2 yields nearly deterministic $I_{n}$ and $N_{\mathrm{Max}}$ under the adopted uncertainty model because it explicitly targets a near-constant normal reaction through fixed-angle scaling, whereas mode 3 introduces additional adaptive regulation that preserves low contact while allowing controlled variability in transient regimes.

Non-parametric statistical testing on the Monte Carlo samples confirmed clear separability between modes for all metrics. Kruskal–Wallis tests were significant for $E_{r}$, $E_{r\mathrm{Max}}$, $I_{n}$, and $N_{Max}$, and all pairwise Mann–Whitney comparisons remained significant after Holm correction (see Table 5), with large effect sizes (Cliff’s δ) for the tracking metrics and near-complete distribution separation for the contact-load metrics. Notably, the only moderate effect size was observed for $E_{r\mathrm{Max}}$ between modes 2 and 3, consistent with the partial overlap seen in Figure 8b, while still remaining highly significant under multiple-comparison correction.

### 3.2 Trade-off analysis between tracking and contact load

To explicitly characterize the tuning trade-off between trajectory tracking accuracy and contact load, we performed a simulation-based parameter sweep of the contact-aware controller (mode3) and constructed a Pareto frontier in the objective space $\left(E_{r},N_{\max}\right)$. The sweep covered a two-dimensional grid of controller settings: the normal-load target $N^{\setminus *}$ (25 values spanning $2\times 10^{-7}$ to $2\times 10^{-6}N$) and the proportional gain $K_{p}$ of the inner reaction/normal-load loop (12 values spanning $1.5\times 10^{-6}$ to $7\times 10^{-6}$), yielding 300 candidate tunings. For each tuning, we simulated the same reference trajectory and computed the same performance metrics defined above; the trade-off was then summarized by retaining only non-dominated solutions (Pareto-optimal points), i.e., those for which no other tuning simultaneously achieved lower $E_{r}\;$ and lower $N_{\max}$.

**Figure 9:**
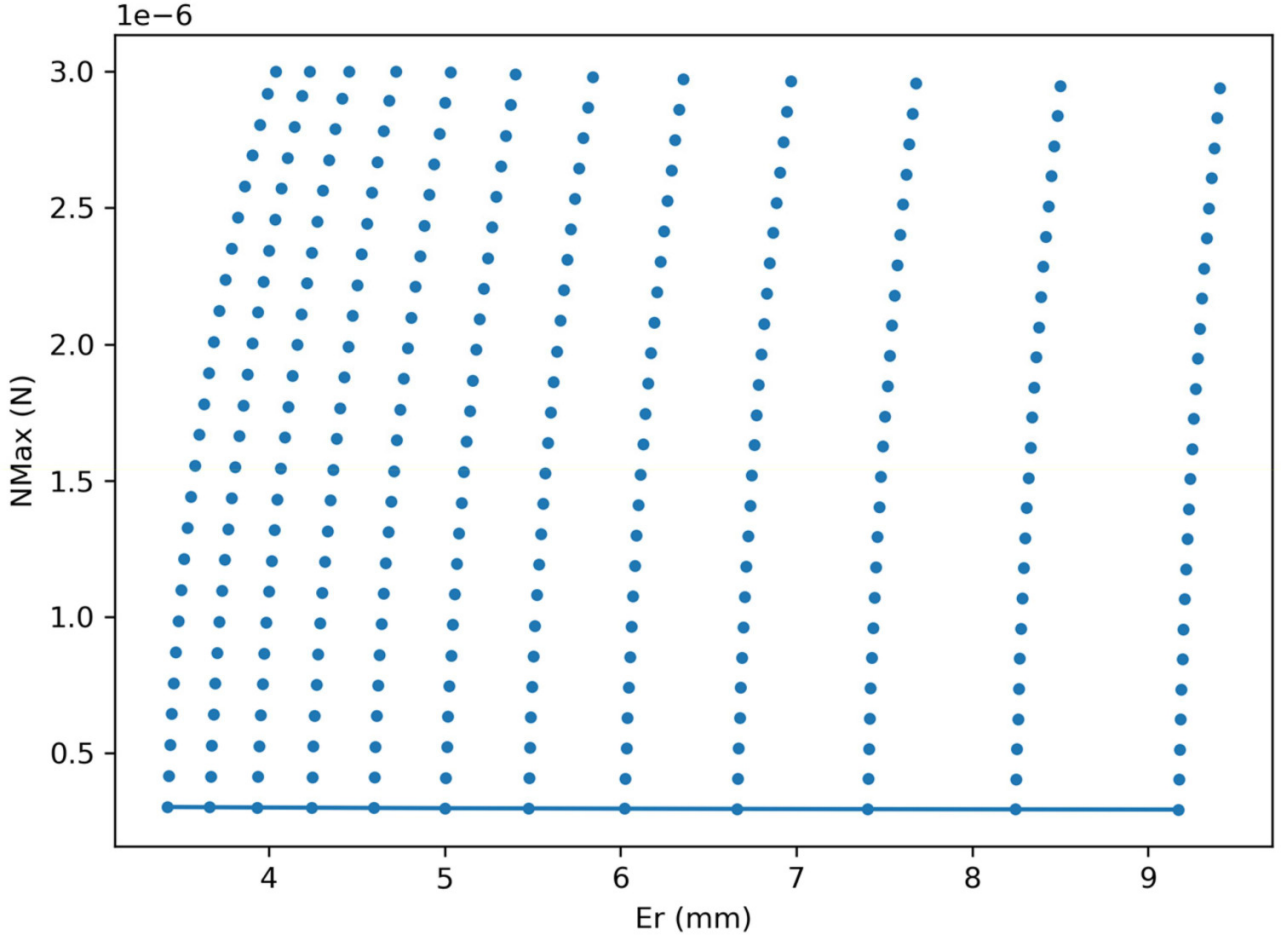
Pareto trade-off between tracking accuracy and contact load (simulation-based sweep). Each point corresponds to one controller tuning in a two-parameter grid sweep over the normal-load target $N^{\setminus *}$ and the inner-loop proportional gain $K_{p}$ (300 candidates). The Pareto frontier highlights the non-dominated tunings in the $\left(E_{r},N_{\max}\right)$ plane, revealing the achievable trade-off between RMS tracking error and peak normal reaction under the same reference trajectory.

Figure 9 shows the resulting scatter and the extracted Pareto frontier. Within the scanned operating range, the Pareto-optimal set concentrated at the lowest $N^{\setminus *}$ explored, indicating that increasing the normal-load target did not provide a compensating reduction in tracking error. Along the frontier, increasing $K_{p}$ improved tracking accuracy (e.g., $E_{r}$ decreased from ∼9.17mm to ∼3.42mm) at the cost of only a modest increase in peak contact load (from $∼2.94\times 10^{-7}\;N$ to $∼3.03\times 10^{-7}\;N$). Overall, this trade-off analysis supports that, for the considered conditions, improving tracking through more aggressive inner-loop tuning does not require a proportional increase in wall loading; instead, the achievable performance lies on a narrow frontier where both objectives remain largely aligned.

### 3.3 Sensitivity to non-newtonian effects

To address the concern that biologically relevant fluids can exhibit shear-thinning behavior, we performed a first-order rheology sensitivity analysis by modifying only the drag term while keeping the remaining model structure, contact/friction parameters, and controller settings unchanged. In the nominal Newtonian case, viscous drag is modeled as $F_{d}=\mu_{F}v$. In the shear-thinning surrogate, we used a velocity-dependent effective coefficient

$$\mu_{\mathrm{eff}}=\mu_{F}{\left(\frac{\max(|v|,v_{0})}{v_{0}}\right)}^{n-1}$$ (11)

with n=0.7 and $v_{0}=10^{-3}\;m\;s^{-1}$ to preserve numerical stability near zero velocity, thus providing a pragmatic “first-order” proxy for shear-thinning without claiming CFD- or lubrication-level fidelity.

**Figure 10:**
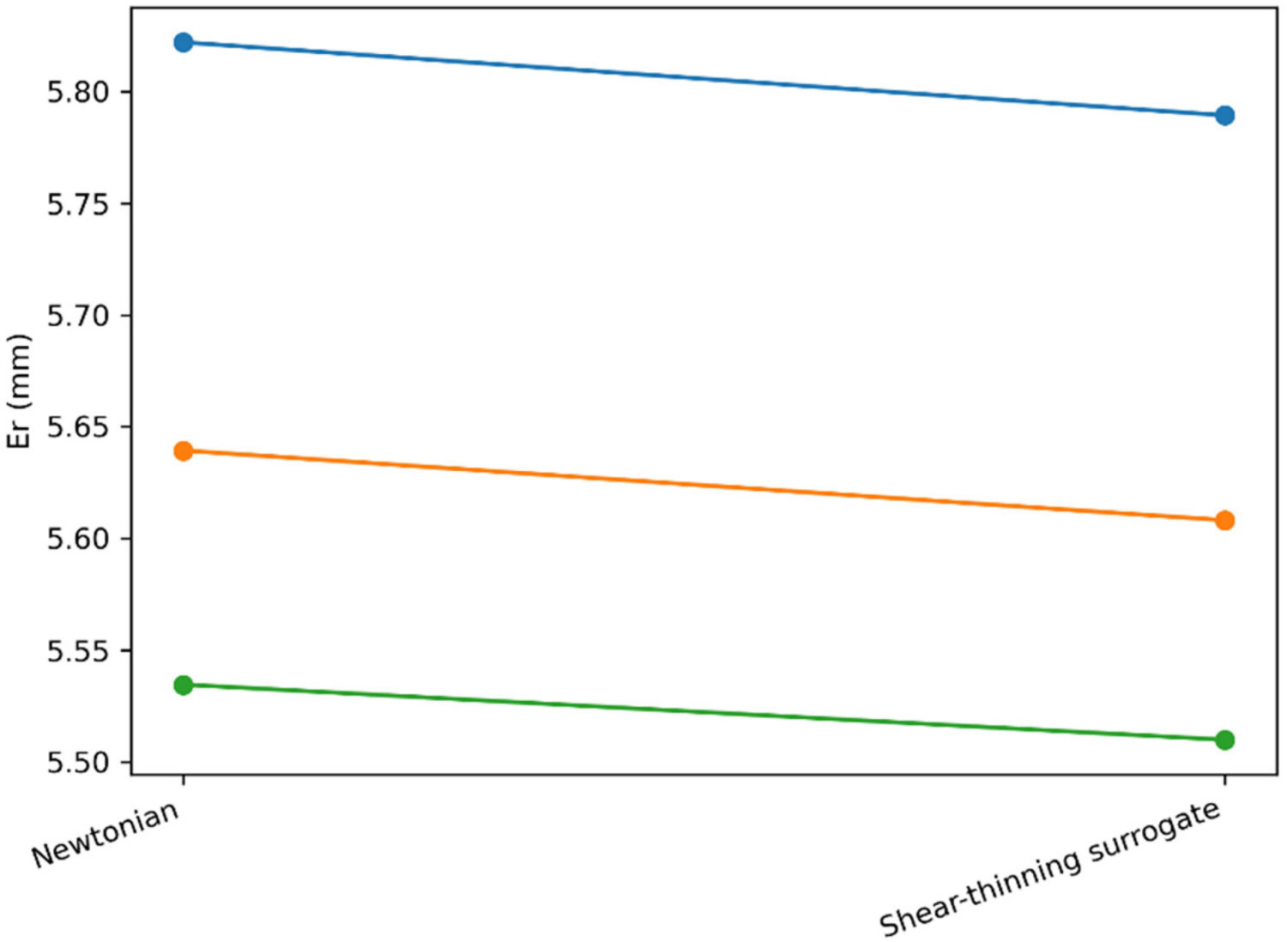
Sensitivity to non-newtonian effects using a shear-thinning surrogate in the drag term.

As shown in Figure 10, switching from a Newtonian drag to the shear-thinning surrogate produces only minor changes in tracking performance and contact-load metrics across the three control modes. Table 6 summarizes the numerical differences: the maximum absolute variation observed across $\;E_{r}$, $E_{r,\max}$, $I_{n}$, and $N_{\max}$remained below ∼0.75% for all modes. Importantly, the qualitative conclusions are preserved: the ranking among the three strategies is unchanged, with mode 3 maintaining the best tracking performance while simultaneously yielding the lowest contact load. Overall, within the scope of this surrogate (i.e., without full lubrication/CFD modeling and without particulate effects), the reported advantages of contact-aware control appear insensitive to moderate shear-thinning behavior.

**Table 6:** Sensitivity to a shear-thinning surrogate (relative to newtonian drag).

Control mode	Er (mm) newtonian → shear-thinning (*Δ*%)	ErMax (mm) newtonian → shear-thinning (*Δ*%)	In (*N*²·s) Newtonian → -thinning (*Δ*%)	NMax (*N*) newtonian → shear-thinning (*Δ*%)
mode1	5.822 → 5.789 (−0.56%)	11.945 → 11.921 (−0.20%)	6.19 × 10^−11^ → 6.17 × 10^−11^ (−0.40%)	2.27 × 10^−6^ → 2.25 × 10^−6^ (−0.74%)
mode2	5.639 → 5.608 (−0.55%)	10.877 → 10.862 (−0.13%)	4.34 × 10^−11^ → 4.34 × 10^−11^ (+0.00%)	1.9 × 10^−6^ → 1.9 × 10^−6^ (+0.00%)
mode3	5.535 → 5.510 (−0.45%)	10.522 → 10.507 (−0.14%)	1.05 × 10^−11^ → 1.05 × 10^−11^ (−0.34%)	1.21 × 10^−6^ → 1.21 × 10^−6^ (−0.12%)

## 4 Discussion

### 4.1 Main findings and translational relevance

This work demonstrates that explicit modeling and closed-loop regulation of wall contact forces are essential for high-performance and safe navigation of a soft, magnetically actuated microrobot in a confined lumen. The proposed contact-aware control architecture, which combines force estimation with magnet-orientation optimization, materially improves both tracking quality and safety metrics. Across the three control modes, the RMS tracking error decreased from 4.8 mm (no force control) to 2.1 mm (force + angle control: −56%), the peak tracking error fell from 14.6 mm to 8.0 mm (−45%), the integrated performance index (In) dropped from 4.4 × 10^−^¹⁰ to 1.6 × 10^−^¹⁰ (−64%), and the peak normal reaction force reduced from 2.0 × 10^−^⁶ N to 0.8 × 10^−^⁶ N (−60%). In a biomedical context, attenuating normal loads at the wall–device interface is central to atraumatic endoluminal interaction in small ducts and distal branches; our results establish magnet angle optimization as a safety-relevant control degree of freedom for managing contact (1, 4, 5, 34).

Simulation-based robustness and statistical significance. Beyond the nominal-case improvements reported above, we quantified robustness under parametric uncertainty using a simulation-based Monte Carlo protocol. The resulting distributions and summary statistics show that the performance ranking is preserved under uncertainty: mean RMS tracking error decreased from 5.81 ± 0.12 mm (mode1) to 5.61 ± 0.04 mm (mode2, −3.5%) and 5.53 ± 0.02 mm (mode3, −5.0%); peak tracking error decreased from 11.96 ± 0.35 mm to 10.86 ± 0.22 mm (−9.3%) and 10.57 ± 0.32 mm (−11.6%); the integrated load surrogate In decreased from (6.24 ± 0.52) × 10^−^¹¹ to 4.33 × 10^−^¹¹ (mode2, −30.5%) and (1.04 ± 0.09) × 10^−^¹¹ (mode3, −83.3%); and peak normal load decreased from (2.24 ± 0.08) × 10^−^⁶ N to (1.87 ± 0.00) × 10^−^⁶ N (mode2, −16.6%) and (1.21 ± 0.13) × 10^−^⁶ N (mode3, −45.9%). Non-parametric testing confirms that inter-mode differences are statistically significant for all metrics, with moderate-to-very-large effect sizes (Cliff's *δ* = 0.50–1.00 depending on the metric and comparison). Collectively, these results substantiate that the benefit of contact-aware control is not limited to a single tuned trajectory or nominal parameter set, but persists under plausible uncertainty in friction/drag and measurement noise.

### 4.2 Alignment with low-Re hydrodynamics and contact-aware control

At micro- to millimeter scales, viscous-dominated dynamics constrain feasible gaits and magnify the impact of wall interactions (10–13). Recent quantitative studies in blood analogs underscore how drag and resistance grow with hematocrit and geometry, stressing the need for explicit modeling of fluid forces and contact mechanics in the controller (34, 35, 37). By embedding contact terms and frictional effects in the model and closing the loop with force and position estimation, the present system directly targets the dominant error sources reported in the literature for confined navigation. The observed 60% reduction in peak normal force dovetails with these insights, indicating that force-aware magnet steering can mitigate sticking and off-axis drift frequently seen in narrow lumens. (34, 35, 37). We performed a parametric scan over controller settings and constructed the feasible set in the {Er, NMax} plane (Figure 9). The scan spans Er ≈ 3.4–9.4 mm and NMax ≈ 0.29–3.0 μN, showing that within this operating regime the two objectives are only weakly coupled (*ρ* ≈ 0.05), i.e., improving tracking does not inherently require higher peak contact load. The lower-left boundary concentrates near configurations that simultaneously reduce error and contact load, supporting the interpretation that (i) explicit normal-load regulation acts as a safety constraint and (ii) magnet-orientation optimization expands the feasible region toward safer navigation without sacrificing tracking performance.

### 4.3 Comparative positioning vs. state-of-the-art magnetic systems

Electromagnet arrays and MRI/superconducting platforms have long been used to sculpt magnetic energy landscapes for steering (3, 16–18), with control strategies ranging from linear optimal control and observers to MPC and learning-based methods (19–21, 30, 38). In contrast, our permanent-magnet approach emphasizes portability and force density while elevating contact-aware objectives to first-class citizens in the control law. Compared with prior works that often simplify wall contact or treat it as a perturbation (22), we show that including contact explicitly and optimizing magnet orientation yields tangible improvements in tracking and load reduction—effects that are clinically meaningful for soft-tissue safety (22, 38).

Prior magnetic navigation systems have demonstrated high-accuracy trajectory tracking using permanent magnets or electromagnetic actuation with observer-based control, often reporting tracking errors as the primary performance endpoint. For example, five-degree-of-freedom permanent-magnet manipulation for capsule-scale devices has shown stable closed-loop control under sensing and update-rate constraints comparable to vision-based systems (2). In vascular-phantom settings, magnetically controlled soft microrobots have also been used to steer tools through three-dimensional anatomically inspired geometries, emphasizing reachability and steerability in branched networks (32). Observer-based magnetic control strategies can further achieve very small tracking errors in controlled laboratory setups (33). However, across these representative studies, wall-contact loads are commonly treated as disturbances or qualitative failure modes, and quantitative reporting of normal reaction forces as a first-class safety metric is still uncommon. In contrast, the present work explicitly models contact and friction, regulates the wall-normal interaction through closed-loop force estimation, and reports normal-load metrics (NMax, In) alongside tracking error, supported by an uncertainty-aware Monte Carlo robustness analysis (*n* = 500) and nonparametric statistical testing.

### 4.4 Clinical plausibility and routes to translation

Two near-term scenarios illustrate applicability: (i) localized drug delivery in sub-millimeter ducts and tortuous vasculature where minimizing normal forces reduces abrasion risk; (ii) gentle micromanipulation (e.g., clot nudging or micro-biopsy) where positional accuracy and controlled contact are paramount. Recent demonstrations of magnetic soft microfiberbots for embolization (36) and the broader clinical push for microrobotic interventions (34) support the therapeutic value of precise, low-load navigation. Our sim–bench agreement and the quantified reductions in error and contact load provide a methodological foundation for developing safe navigation strategies. This aligns with the emphasis in clinical-facing reviews on the importance of controlled interaction for translational success (34, 36). Direct validation in anatomical phantoms and with biological tissues is the necessary next step.

Importantly, these results should be interpreted as a bench-validated control principle rather than a clinically ready system. The present channel is rigid acrylic and the normal force is model-estimated (not directly measured), so we do not claim quantitative safety margins relative to mucosal injury thresholds, which depend on tissue compliance, lubrication, and exposure time. Likewise, clinical translation would require (i) anatomical-scale phantoms and tissue analogs, (ii) clinically compatible sensing/imaging for state estimation, and (iii) a dedicated safety assessment of static magnetic fields in realistic clinical environments (including constraints posed by implants and ferromagnetic objects). These elements define the next translational stage once the contact-aware control methodology is established.

### 4.5 Novel contributions relative to prior art

First, the study integrates a deformable soft body (silicone with embedded magnetic particles) and explicit wall-contact modeling within a closed-loop architecture that jointly optimizes force and magnet orientation—a combination less explored in prior electromagnet-centric works (2, 35, 38). Second, the quantitative gains (−56% RMS error; −60% peak normal force) move beyond qualitative trajectory following to safety-relevant metrics, speaking directly to device-level performance. Third, the portable permanent-magnet actuation highlights a clinical footprint complementary to large, cost-intensive systems, aligning with ongoing efforts toward deployable magnetic navigation solutions. (35, 38).

### 4.6 Agreement between simulation and experiment

Combined time-series showed qualitative agreement between model and bench, with residual deviations attributable to material and surface heterogeneity (acrylic channel roughness, MO coating variability), parameter identification errors, and unmodeled viscoelastic effects of the fluid–wall system—factors consistently reported to perturb microrobot dynamics in confined environments (30, 37, 40). Our findings echo recent contact-mechanics studies in soft robotics showing that silicone friction coefficients and temperature/pressure dependencies must be represented to predict load transfer accurately under sliding/indentation, especially in soft–hard interfaces (40).

### 4.7 Comparison to recent clinical-proximal demonstrations

While shape-reconfigurable microfiberbots (36) emphasize embolic manipulation with multi-modal actuation, and catheter-scale systems focus on steerable tips (4, 5), our contribution targets the force/trajectory regulation problem for untethered soft bodies in narrow lumens using permanent-magnet steering. The complementarity is clear: advances in device materials and reconfiguration need to be matched with contact-aware controllers that keep normal loads low and tracking errors bounded—particularly relevant as clinical pathways for microrobotic delivery mature (34, 35).

### 4.8 Limitations and future works

(1) *in-vitro* bench with acrylic channels and camera-based tracking; real tissues exhibit compliance, viscoelasticity, and surface chemistry that alter contact and drag. (2) Newtonian fluid approximation; blood's hematocrit-dependent rheology and particulate interactions change resistance and stability (37). (3) Small errors in field/gradient maps propagate to force estimation and control optimality (22). (4) The controller design is primarily linear in its outer-loop logic; handling intermittent contact and adhesion may benefit from nonlinear/MPC or learning-based strategies with explicit contact constraints (38).

Future works can: (i) Conducting a large number of experiments and performing detailed statistical analysis on all key metrics. (ii) Testing in phantoms with viscoelastic, tissue-like walls and non-Newtonian, cell-laden fluids to benchmark performance (tracking error, normal force) under more physiologically realistic conditions (37). (iii) Incorporating data-driven or tribology-informed models for silicone-tissue contact to improve the prediction of friction and adhesion forces (40). (iv) Extending the framework to full 3D tracking and control, potentially integrated with medical imaging modalities, requiring enhanced state estimation and field calibration. (v) Implementing and testing nonlinear MPC or adaptive/learning-based controllers explicitly designed to handle contact-state transitions and model uncertainties while guaranteeing safety constraints (38). (vi) As the core control methodology matures, future research beyond this study will need to address system-level challenges for clinical translation, including the design of clinically viable actuation systems, integration with clinical imaging and workflows, and ensuring safety and compatibility in a medical environment (34, 35).

## 5 Conclusion

This study introduced a contact-aware control framework for magnetically actuated, soft deformable microrobots navigating confined lumens. The approach combines an explicit dynamic model of viscous drag, nonlinear friction, and viscoelastic wall contact with a closed-loop architecture that couples position regulation to model-based force estimation and magnet-orientation optimization. In the nominal scenario, the force-plus-angle strategy delivered substantial improvements in both tracking and contact-load metrics relative to baseline control modes, while maintaining qualitative agreement between simulation and bench trajectories.

To strengthen the evidence base beyond a single nominal setting, we additionally performed a simulation-based robustness and inference layer. A Monte Carlo protocol (*n* = 500 trials per mode) demonstrated that the performance ordering across control modes is preserved under plausible uncertainty in drag/friction parameters and measurement noise, with consistent reductions in tracking error and contact load for the contact-aware strategies. Non-parametric statistical testing further supported that inter-mode differences are significant across key metrics, with moderate-to-large effect sizes. A trade-off analysis in the $\left\{E_{r},N_{\max}\right\}$ plane showed that, in the scanned operating regime, improved tracking does not inherently require higher peak contact forces, and that magnet orientation provides an effective degree of freedom for moving toward safer operating points. Finally, a first-order sensitivity study using a shear-thinning surrogate indicated that the main conclusions are not materially altered by moderate non-Newtonian behavior at the level represented by this simplified drag modification.

Overall, the results support the central claim that force-aware steering is not merely a tracking enhancement but a safety-relevant control capability for endoluminal navigation, enabling simultaneous reduction of trajectory deviations and contact loads. The permanent-magnet implementation serves as a practical research platform emphasizing simplicity and high force density; importantly, the control principle is transferable to other actuation systems, including electromagnetic coil arrays. Key next steps toward clinical translation include repeated-trial experimental campaigns with full time-series logging for direct experimental statistics, validation in biomimetic phantoms with compliant tissue-like walls and non-Newtonian, cell-laden fluids, improved magnetic field/gradient calibration, extension to full 3D state estimation and image-guided navigation, and systematic evaluation of failure modes and safety constraints relevant to clinical environments.

## Data Availability

The raw data supporting the conclusions of this article will be made available by the authors, without undue reservation.

## References

[1] Nelson BJ Kaliakatsos IK Abbott JJ. Microrobots for minimally invasive medicine. Annu Rev Biomed Eng. (2010) 12(1):55–85. 10.1146/annurev-bioeng-010510-103409 20415589

[2] Mahoney AW Abbott JJ. Five-degree-of-freedom manipulation of an untethered magnetic device in fluid using a single permanent magnet with application in stomach capsule endoscopy. Int J Rob Res. (2016) 35(1–3):129–47. 10.1177/0278364914558006

[3] Xu T Zhang L Sitti M. Magnetic actuation based motion control for microrobots: an overview. Micromachines (Basel). (2015) 6(9):1346–64. 10.3390/mi6091346

[4] Hwang J Kim J Choi H. A review of magnetic actuation systems and magnetically actuated guidewire- and catheter-based microrobots for vascular interventions. Intell Serv Robot. (2020) 13(1):1–14. 10.1007/s11370-020-00311-0

[5] Pittiglio G Ricotti L Menciassi A Beccai L. Patient-specific magnetic catheters for atraumatic autonomous endoscopy. Soft Robot. (2022) 9(6):1120–33. 10.1089/soro.2021.0090 35312350 PMC9805888

[6] Chen H Chen M Sun M Metin S Wang Q. An overview of micronanoswarms for biomedical applications. ACS Nano. (2021) 15(10):15625–44. 10.1021/acsnano.1c07363 34647455

[7] Kim Y Zhao X. Magnetic soft materials and robots. Chem Rev. (2022) 122(5):5317–64. 10.1021/acs.chemrev.1c00481 35104403 PMC9211764

[8] Schmidt CK Medina-Sánchez M Edmondson RJ Schmidt OG. Engineering microrobots for targeted cancer therapies from a medical perspective. Nat Commun. (2020) 11(1):5618. 10.1038/s41467-020-19322-7 33154372 PMC7645678

[9] Felfoul O Martel S Côté J. Magneto-aerotactic bacteria deliver drug-containing nanoliposomes to tumour hypoxic regions. Nat Nanotechnol. (2016) 11(11):941–7. 10.1038/nnano.2016.137 27525475 PMC6094936

[10] Edd J Maggi A Belcher A Sitti M. Biomimetic propulsion for a swimming surgical micro-robot. *In* Proceedings of the 2003 IEEE/RSJ International Conference on Intelligent Robots and Systems (IROS 2003) (2003). Vol. 3, p. 2583–8). IEEE. 10.1109/IROS.2003.1249259

[11] Choi H Park JO Park S Song S. Two-dimensional locomotion of a microrobot with a novel stationary electromagnetic actuation system. Smart Mater Structu. (2009) 18(11):115017. 10.1088/0964-1726/18/11/115017

[12] Yu C Kim T Kim S Park S. Novel electromagnetic actuation system for three-dimensional locomotion and drilling of intravascular microrobot. Sens Actuators, A. (2010) 161(1–2):297–304. 10.1016/j.sna.2010.04.037

[13] Mathieu J-B Beaudoin G Martel S. Method of propulsion of a ferromagnetic core in the cardiovascular system through magnetic gradients generated by an MRI system. IEEE Trans Biomed Eng. (2006) 53(2):292–9. 10.1109/TBME.2005.862570 16485758

[14] Abbott JJ Nagy Z Mark M Nelson BJ. Modeling magnetic torque and force for controlled manipulation of soft-magnetic bodies. IEEE Trans Robot. (2007) 23(6):1247–52. 10.1109/TRO.2007.910775

[15] Pawashe C Floyd S Sitti M. Modeling and experimental characterization of an untethered magnetic micro-robot. Int J Rob Res. (2009) 28(8):1077–94. 10.1177/0278364909341413

[16] Gillies GT Ritter RC Grady MS Howard MA Broaddus WC. Magnetic manipulation instrumentation for medical physics research. Rev Sci Instrum. (1994) 65(3):533–62. 10.1063/1.1145242

[17] Quate EG Wika KG Lawson MA Gillies GT Ritter RC Grady MS Goniometric motion controller for the superconducting coil in a magnetic stereotaxis system. IEEE Trans Biomed Eng. (1991) 38(9):899–905. 10.1109/10.83610 1743738

[18] Takeda S Ohtsuka K Matsunaga T Hashimoto Y. Development of magnetically targeted drug delivery system using superconducting magnet. J Magn Magn Mater. (2007) 311(1):367–71. 10.1016/j.jmmm.2006.10.1195

[19] Tamaz S Gourdeau R Chanu A Mathieu JB Martel S. Real-time MRI-based control of a ferromagnetic core for endovascular navigation. IEEE Trans Biomed Eng. (2008) 55(7):1854–63. 10.1109/TBME.2008.919720 18595804

[20] Choi J Park J Park S Song S. Position stabilization of microrobot using pressure signal in pulsating flow of blood vessel. 2010 IEEE Sensors IEEE. (2010). p. 723–6. 10.1109/ICSENS.2010.5690046

[21] Arcese L Fruchard M Ferreira A. Adaptive controller and observer for a magnetic microrobot. IEEE Transactions on Robotics. (2013) 29(4):1060–7. 10.1109/TRO.2013.2257581

[22] Edelmann J Petruska AJ Nelson BJ. Magnetic control of continuum devices. Int J Rob Res. (2017) 36(1):68–85. 10.1177/0278364916683443

[23] Xu H Medina-Sánchez M Magdanz V Schwarz L Schmidt OG. Sperm micromotors for cargo delivery through flowing blood. ACS Nano. (2020) 14(3):2982–93. 10.1021/acsnano.9b07851 32096976

[24] Lucarini G Beccai L Menciassi A. Navigation of magnetic microrobots with different user interaction levels. IEEE Trans Autom Sci Eng. (2014) 11(3):818–27. 10.1109/TASE.2014.2317232

[25] Malchikov AV Yatsun SF Ryapolov PA Zharov VE. An experimental stand for studying the motion of magnetically active objects under the influence of the magnetic field of a movable magnet. Robot Tech Cybernetics. (2024) 12(4):270–9. 10.31776/RTCJ.12404

[26] Malchikov AV Yatsun SF Ryapolov PA Sokolov EA. Studying the controlled motion of a magnetically active object in a viscous medium. Bull Russ Acad Sci Phys. (2025) 89(7):1111–7. 10.1134/S106287382571178X

[27] Malchikov A Ryapolov P. A study of the magnetically active objects motion in a closed channel under the influence of magnetic force. In 2024 International Russian Automation Conference (RusAutoCon) IEEE. (2024). p. 409–14. 10.1109/RusAutoCon61949.2024.10694236

[28] Lee L-H (ed) Fundamentals of Adhesion. New York, NY: Springer Science & Business Media (2013).

[29] Choi I Lim C. Low-velocity impact analysis of composite laminates using linearized contact law. Compos Struct. (2004) 66:125–32. 10.1016/j.compstruct.2004.04.030

[30] Arcese L Fruchard M Ferreira A. Endovascular magnetically guided robots: navigation modeling and optimization. IEEE Trans Biomed Eng. (2012) 59(4):977–87. 10.1109/TBME.2011.2181508 22203703

[31] Malchikov A Yatsun S Ryapolov P. Investigation of a contactless control system for micro-object based on magnetically active materials. Proceedings of the 2025 Conference of Young Researchers in Electrical and Electronic Engineering IEEE. (2025). p. 1184–8.

[32] Jeon S Hoshiar AK Kim K Lee S Kim E Lee S A magnetically controlled soft microrobot steering a guidewire in a three-dimensional phantom vascular network. Soft Robot. (2019) 6:54–68. 10.1089/soro.2018.0019 30312145 PMC6386781

[33] Lu J Liu Y Huang W Bi K Zhu Y Fan Q. Robust control strategy of gradient magnetic drive for microrobots based on extended state observer. Cyborg Bionic Syst. (2022) 2022:9835014. 10.34133/2022/9835014 36320320 PMC9619236

[34] Iacovacci J Serafini MS Avuzzi B Badenchini F Cicchetti A Devecchi A Intestinal microbiota composition is predictive of radiotherapy-induced acute gastrointestinal toxicity in prostate cancer patients. EBioMedicine*.* (2024)106:105246. 10.1016/j.ebiom.2024.105246 39029427 PMC11314862

[35] Zhou Y Xing F Zhao C Clinical observation on treatment of refractory ascites in liver cirrhosis by modified tuduzi buqi decoction combined with western medicine conventional therapy. Shanghai J Tradit Chin Med. (2021) 55:67–9, 76.

[36] Liu Y Yi JH Wang PY Fu P Kang Y Wang T Safety evaluation of extracorporeal shockwave lithotripsy for pancreatic stones: Experience based on a large chronic pancreatitis cohort. Dig Liver Dis*.* (2025) 57(2):417–26. 10.1016/j.dld.2024.08.043 39261265

[37] Wu ZS Lee WC Chancellor MB Wang HJ Huang CC Chuang YC. Extracorporeal shock wave therapy modulates miRNA expression, reduces inflammation, and improves liposome retention in a rat model of cyclophosphamide-induced cystitis - experimental studies. Int J Surg. (2024) 110(11):6869–72. 10.1097/JS9.0000000000001961 39037744 PMC11573052

[38] Jiang Y Wang F Wang K Zhong Y Wei X Wang Q Engineered exosomes: a promising drug delivery strategy for brain diseases. Curr Med Chem. (2021) 29(17):3111–24. 10.2174/0929867328666210902142015 34477508

[39] Hou J Xu F Du H Liu J Li N. Efficacy and safety of the surgical treatments for lower calyceal stones: a systematic review and network meta-analysis. Int J Surg. (2023) 109(3):383–8. 10.1097/JS9.0000000000000062 36906759 PMC10389214

[40] Berthold EC Kamble SH Raju Kanumuri SR Kuntz MA Senetra AS Chiang Y-H Comparative pharmacokinetics of commercially available cannabidiol isolate, broad-spectrum, and full-spectrum products. Eur J Drug Metab Pharmacokinet*.* (2023) 48:427–35. 10.1007/s13318-023-00839-3 37337087

